# Functional relevance of *in vivo* half antibody exchange of an IgG4 therapeutic antibody-drug conjugate

**DOI:** 10.1371/journal.pone.0195823

**Published:** 2018-04-19

**Authors:** Peter Herbener, Kurt Schönfeld, Martin König, Matthias Germer, Jude M. Przyborski, Katrin Bernöster, Jörg Schüttrumpf

**Affiliations:** 1 Corporate Research & Development, Biotest AG, Dreieich, Germany; 2 Parasitology, Faculty of Biology, Philipps University Marburg, Marburg, Germany; 3 Corporate Project & Portfolio Management, Biotest AG, Dreieich, Germany; Mie University Graduate School of Medicine, JAPAN

## Abstract

An increasing number of monoclonal antibodies and derivatives such as antibody-drug conjugates (ADC) are of the IgG1 and IgG4 isotype with distinct structural and functional properties. In cases where antibody-mediated cytotoxicity is not desired, IgG4 is often used, as its Fc region is relatively poor at inducing antibody-dependent cell-mediated or complement-dependent cytotoxicity. IgG4 ADCs with highly cytotoxic drugs against proliferating target cells but which lack or have diminished antibody effector functions against quiescent cells may have a favorable safety profile compared to IgG1. Another unique property of the IgG4 subclass is the capability to exchange half antibodies *in vivo* creating randomly bispecific antibodies. To investigate the functional properties of process-derived antibody species, and determine the influence of shuffling on the therapeutic efficacy, several model antibodies on the basis of the anti-CD138 antibody-drug conjugate BT062 (Indatuximab ravtansine) were generated: (I) A wild type nBT062, (II) a stable nBT062 comprising mutations to prevent half-antibody exchange, (III) a half nBT062 lacking covalent binding between two heavy chains and (IV) a stabilized, bispecific nBT062-natalizumab antibody with a second, monovalent specificity against CD49d. All nBT062 model variants were capable of CD138-specific binding and antigen-mediated internalization into cells. Furthermore, all nBT062 models inhibited tumor growth *in vitro* after conjugation with the maytansinoid DM4. The *in vivo* effects of the different molecular variants were assessed in the MAXF1322 xenograft model. The bispecific nBT062-natalizumab-DM4 demonstrated the least efficacy and was only moderately active even without the co-administration of a human IgG preparation. Wild type, stable and half nBT062-DM4 models demonstrated great anti-tumor activities. The efficacy of wild type and half nBT062-DM4 was reduced in the presence of IgG, while stable nBT062-DM4 was only marginally influenced. These pre-clinical data demonstrate the advantage of introducing half-antibody exchange-preventing mutations into therapeutic IgG4-based antibody drug-conjugates.

## Introduction

The choice between the immunoglobulin gamma (IgG) 1, IgG2 and IgG4 isotypes during the discovery phase of monoclonal antibodies involves careful consideration of the intended *in vivo* effect. Antibody therapeutics can be different in their mode of action. Mechanisms including neutralizing soluble mediators, binding to cells and killing them, and regulating cell functions can be optimized. The selection of the variable antigen binding domain includes criteria such as target specificity, affinity, and pharmacology. The choice of the constant fragment crystallizable (Fc) region focuses on whether specific effector functions are required for best efficacy or undesirable for safety reasons, and additionally on the need for a suitable half-life in the patient.

**Fig 1:**
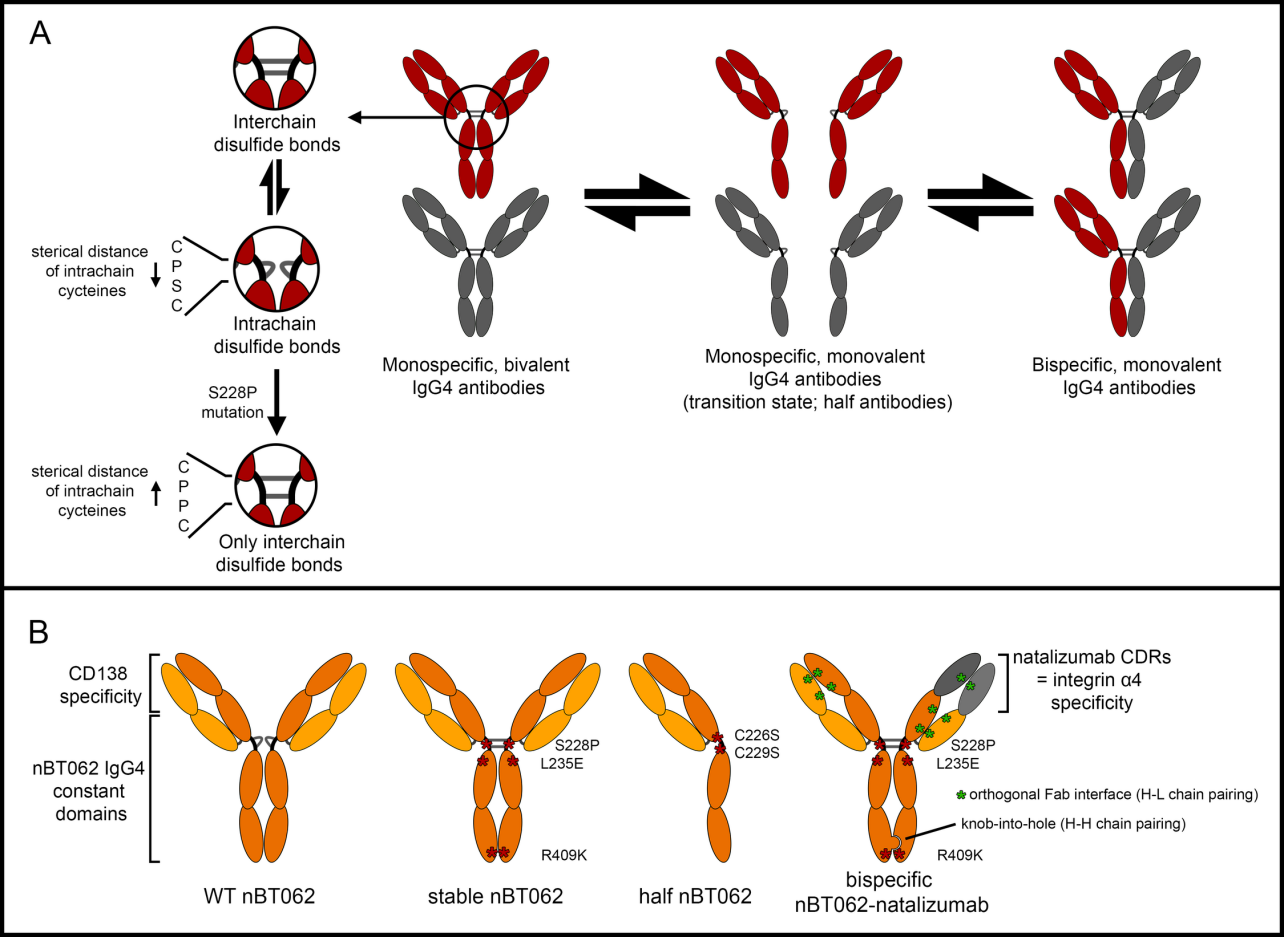
Schematic half antibody exchange and respective nBT062 model antibodies. (A) Overview of *in vivo* IgG4 half antibody exchange mainly driven by the potential to form either interchain or intrachain disulfide bonds in the hinge region. A S228P mutation increases the sterical distance of the two cysteines preventing intrachain disulfide bonds. (B) Generated nBT062 model antibodies: Wild type (WT) nBT062, stable nBT062, half nBT062 and bispecific nBT062-natalizumab. Respective mutations are indicated.

In contrast to other IgGs, IgG4 antibodies exhibit only weak Fcγ-receptor and C1q binding necessary for antibody-dependent cell-mediated cytotoxicity (ADCC) and complement-dependent cytotoxicity (CDC) [1,2]. In addition, they are the only Ig class capable of exchange half antibodies (heavy (H) + light (L) chain) *in vivo*, which results in monovalent, bispecific antibodies [3,4]. IgG1 molecules harbor a CPPC motif within the hinge region. These two prolines create steric hindrance between the cysteines that IgG1 half antibodies are only able to form interchain disulfide bonds with a second IgG1 half-antibody. On the contrary, IgG4 antibodies have a CPSC motif resulting in a more flexible hinge region with a decreased distance between the two cysteines, which allows the formation of either inter- or intrachain disulfide bonds (Fig 1A) [5,6]. R409 is the crucial residue at the CH3-CH3 interface, weakening the non-covalent association between these domains and allowing half antibody exchange [7,8]. By mutating either S228P or R409K IgG4 antibodies are stabilized, while introduction of P228S and K409R mutations in IgG1 antibodies enable half-antibody shuffling [7].

CD138 (Syndecan 1) is a transmembrane heparin sulfate proteoglycan and a member of the syndecan proteoglycan family. It is involved in several cellular functions, including cell-cell adhesion, migration, signaling, cell-matrix interactions and proliferation. The latter is mediated by growth factor binding such as fibroblast growth factor 2 and transforming growth factor ß1 [9–13]. CD138 is expressed on epithelial cells and different hematopoietic cells: While antibody producing plasma cells and B cell precursors express CD138, it is absent on circulating mature B cells and CD34-positive stem cells [11,14,15]. However, during malignant hematopoiesis and several solid tumors including breast, pancreas, bladder, lung and prostate cancer expression of CD138 is highly upregulated [11,16,17].

Indatuximab ravtansine, also referred to as BT062, is an antibody drug conjugate composed of the CD138-specific antibody nBT062 (naked BT062) chemically linked to the tubulin-binding maytansinoid DM4. nBT062 is a hinge-unmodified human IgG4 chimerized antibody based on the murine B-B4 precursor. B-B4 was shown to bind the linear epitope between residues 90 to 93 of the CD138 core protein [18,19]. BT062 is currently evaluated in several clinical trials in multiple myeloma [20], breast and bladder cancer (EudraCT No.: 2013-003252-20). The proposed mode of action for disulfide bond-based antibody-maytansinoid conjugates includes antigen-binding, internalization by endocytosis and release of the toxic agent within the cell leading to target cell death and beneficial bystander killing of directly neighboring cells via DM4 diffusion [18]. In proliferating cells DM4 inhibits proper microtubule assembly. Thus, cell cycle arrest and apoptosis are induced [21]. Only free, but not antibody-conjugated DM4 is able to pass through plasma membranes [22]. Therefore, BT062 might exhibit no damage to healthy tissues and possess low systemic toxicity. Gemtuzumab ozogamicin and inotuzumab ozogamicin are two FDA approved IgG4-based ADCs. Despite they both contain a hinge-region stabilizing S228P mutation to prevent half antibody exchange [23], a direct influence of this *in vivo* mechanism on the functionality and efficacy on ADCs has never been studied in detail so far.

In this current study four antibody variants wild type (WT) nBT062, stable nBT062, half nBT062, and bispecific nBT062-natalizumab were generated. Natalizumab (Biogen) is a therapeutic IgG4 antibody approved for the treatment of multiples sclerosis and Crohn's disease and binding to the integrin α4 subunit (CD49d). Since it is unstabilized and half-antibody exchange has previously been demonstrated [24], its complementarity determining regions (CDR) were used to design a bispecific model antibody. These four antibody variants were used to investigate the functional activity of potentially *in vivo* derived IgG4 antibodies species and determine the influence of IgG4 shuffling on the efficacy.

## Materials and methods

### Generation of CD138-specific antibodies

The sequence of WT nBT062 was used as basis for the generation of stable nBT062, half nBT062 and bispecific nBT062-natalizumab. All mutations were introduced by full length DNA synthesis at Thermo Fisher Scientific, Germany. The stabilizing amino acid mutations S228P and R409K were introduced to design the stable nBT062 antibody [7]. Additionally a L235E mutation was introduced into this variant for reduction of FcγR binding [25]. The cysteines building up the disulfide bonds of the hinge region were exchanged by serines (C226S + C229S) to create the half nBT062. For the generation of the bispecific nBT062-natalizumab antibody, the available CDR and framework regions of natalizumab H and L chains [26]were aligned to the stable nBT062 sequence to determine the exchange of the respective amino acid sections. Knob and hole technology was used to receive correct heavy chain pairing during co-expression of the two different heavy and light chains [27]. To ensure correct H to L chain pairing, orthogonal antigen-binding fragment (Fab) interfaces were used [28]: The nBT062 Fab of the bispecific nBT062-natalizumab comprises D1R and Q38D mutations in the light chain and Q39K and K62E mutations in the variable region of the heavy chain. Q38R, S176W and L135Y mutations were introduced to the light chain of the natalizumab Fab of bispecific nBT062-natalizumab, while Q39Y, F174G and H172A mutations were applied to the heavy chain. Full length DNA sequences of the model antibodies were synthesized and cloned into pEF mammalian expression vectors. Heavy and light chains of monovalent antibodies WT nBT062, stable nBT062 and half nBT062 were cloned into separate vectors containing either a puromycin or methotrexate (MTX) resistance. Two plasmids carrying the sequences of the bispecific nBT062-natalizumab were cloned as follows: Heavy and light chain sequences of the nBT062 part were both cloned into the pEF vector containing a puromycin resistance, whereat a T2A element [29] and a furin cleaving site were introduced between heavy and light chains for further separation. Heavy and light chains of the natalizumab part, also interrupted by a T2A element and furin recognition site, were cloned into the pEF vector comprising a MTX resistance. The T2A element processes heavy and light chain sequences on the translational level while furin further deletes post-translationally introduced spacer residues.

1x10^6^ FreeStyle CHO-S cells (Life Technology) were co-transfected with 5 μg of each plasmid DNA (10 μg in total for co-expression) using program FF-137 on the 4D nucleofector system (Lonza). For the generation of cell pools stably expressing the antibodies, cells were cultured in MTX/Puromycin containing FreeStyle CHO Expression Medium (Thermo Fisher Scientific) in 24 well plates until populations grew up where genome integration took place. For antibody production, cells were grown in 1L shaking culture at 37°C/ 5% CO_2_/ 125 rpm until viability dropped below 80%. The cells were centrifuged and the antibodies in the supernatant were purified on 5 ml MabSelect Protein A columns (GE Healthcare) using an Äkta Avant 150 System (GE Healthcare).

### Electrophoresis and western blotting

10 μg of purified WT nBT062, stable nBT062, half nBT062, bispecific nBT062-natalizumab and natalizumab were separated by sodium dodecyl sulfate polyacrylamide gel electrophoresis (SDS-PAGE) using a 4–12% Bis-Tris gel (Novex) and MOPS SDS Running buffer. Samples were either separated under reducing (25% NuPAGE LDS Sample Buffer (Thermo Fisher) + 10% NuPAGE Sample Reducing Agent (Thermo Fisher), 10 min at 70°C) or non-reducing (25% NuPAGE LDS Sample Buffer + 25% of 400mM N-ethylmaleimide solution, 30 s at 70°C) conditions. Gels were fixed for 45 min in 37.2% methanol/ 7% acetic acid solution and stained for 3 h in Coomassie brilliant blue solution. Destaining was performed over night in 25% methanol solution.

For isoelectric focusing (IEF), 5 μg of WT nBT062, stable nBT062, half nBT062, bispecific nBT062-natalizumab and natalizumab were mixed with 50% Novex IEF Sample Buffer pH 3–10 (Thermo Fisher Scientific) and applied to a Novex pH 3–10 IEF gel in IEF Anode and Cathode buffer (Thermo Fisher Scientific). Proteins were separated in a stepwise process: 100 V for 60 min, 200 V for 60 min and 500 V for 30 min. Gels were either fixed for 30 min in 12% TCA/ 3.5% sulfosalicylic acid and stained with Coomassie blue or subsequently blotted onto a PVDF membrane. The membrane was blocked over night at 4°C in Odyssey Blocking buffer (Licor) and incubated simultaneously with 1 μg/ml anti-BT062-Biotin (Biotest) and anti-natalizumab-HRP (Bio-Rad) for 2h at room temperature. Binding of anti-idotypic antibodies was visualized after 2 h of simultaneous incubation with anti-HRP-800CW and Streptavidin-680RD (Licor) on Licors Odyssey Imager.

### Cell cultivation

Human cell lines, NCI-H929 (Cat.-No. CRL-9068) and Jurkat (Cat.-No. TIB-152) were obtained from the American Type Culture Collection (ATCC) and murine BaF3 cells (Cat.-No. ACC 300) were obtained from Leibniz-Institut DSMZ (Germany). BaF3-hCD138 cells were generated by lentiviral transduction of BaF3 cells with human CD138 at SIRION Biotech (Germany). All cell lines were cultured at 37°C, 5% CO2 and 100% atmospheric humidity in a Thermo Scientific Incubator. NCI-H929 cells were maintained in RPMI 1640 medium (Lonza) supplemented with 10% fetal bovine serum (FBS, Thermo Fisher Scientific) and 2 mM L-glutamine (Lonza); Jurkat cell medium was additionally supplemented with 10 mM HEPES buffer (Thermo Fisher Scientific) and 1 mM sodium pyruvate (Thermo Fisher Scientific). BaF3 cells were cultured in RPMI 1640 medium supplemented with 10% FBS and 10 ng/ml mouse IL-3 (R&D Systems). Cells were passaged two to three times per week and handled as recommended by the suppliers.

### Antibody labeling with Dylight 488

For direct detection of nBT062 model variants during flow cytometric and fluorescence microscopic internalization experiments, WT nBT062, stable nBT062, half nBT062 and bispecific nBT062-natalizumab were labeled with a Dylight 488 (Dy488) fluorescent dye (Thermo Fisher Scientific) according to the manufacturer's instructions. In addition, natalizumab was labeled as control antibody. In brief, 40 μl of 0.67 M borate buffer were added to 500 μl of each antibody solution in DPBS (2.2 mg/ml). Each resulting solution was transferred into a vial containing the Dy488 dye powder, mixed by vortexing and the labeling reaction was carried out for 60 min in the dark. Unbound dye molecules were removed by the kit-containing spin columns. Antibody concentrations and antibody-to-dye ratios were determined by photometric absorptions measured at 280 and 493 nm wavelength using the following formulas:

$$Protein\;concentration\;\left(\frac{g}{l}\right)=\frac{A_{280}-(A_{max}*CF)}{\varepsilon_{protein}}*DF*m$$

$$ratio\;dye:protein=\frac{A_{max}*DF}{\varepsilon_{Dye}*c_{Protein}}$$

DF (dilution factor) = 10

ε_protein_ = 210 000 for IgG

m (specific mass) = 150000 Da

Amax = 493

CF = 0.147

ε_Dye_ = 70.000

The Dy488 labeling reaction results in dye-to-antibody ratios of 1.92, 2.17, 1.82, and 1.99 for WT nBT062, stable nBT062, half nBT062 and bispecific nBT062-natalizumab, respectively.

### Flow cytometry

In order to analyze the specific binding capacity of the four different nBT062 variants or their respective DM4-conjugates as well as natalizumab, BaF3-hCD138 (CD138+/ CD49d-), Jurkat (CD138-/ CD49d+) or NCI-H929 (CD138+/ CD49d+) cells were incubated with 3-fold serial antibody dilutions. Cells were seeded in 96 well plates (150,000 cells/well) and incubated for 30 min at 4°C in 100 μl antibody solution (5x10^-7^–2.82x10^-12^ molar). Cells were washed twice with PBS/3%FBS and incubated with 10 μg/ml of alexa fluor 647-labelled goat anti-human secondary antibody (Thermo Fisher) for 0.5 h at 4°C. Subsequently, the antibody solution was discarded, cells were washed twice with PBS/3%FBS and analyzed with a FACS Canto II flow cytometer (Beckton Dickinson).

To determine nBT062 variant internalization, NCI-H929 cells (CD138+/ CD49d+) were seeded in 96 well plates (100,000 cells/well). Cells were incubated with 15 μg/ml of either Dylight-488 (Dy488) labeled WT nBT062, stable nBT062, half nBT062 or bispecific nBT062-natalizumab antibodies. Cell surface staining was analyzed after 0.5 h of antibody incubation at 4°C, while internalization was analyzed after 24 h incubation at 37°C. To prevent bispecific nBT062-natalizumab from binding to CD49d, natalizumab was co-incubated with a 50-times excess. Cells were either incubated with Trypsin for 10 min at 37°C to remove CD138 including bounded antibodies or not and washed twice with PBS/3%FBS. Analysis was performed using a FACS Canto II flow cytometer and data evaluation was done with GraphPad Prism 6.1.

### Fluorescence microscopy

To further investigate CD138-mediated internalization of nBT062 variant antibodies, BaF3 (CD138-/ CD49d-) and BaF3-hCD138 (CD138+/ CD49d-) cells were seeded in 96 well plates (150,000 cells/wells) and incubated for 0.5 h at 4°C with 10 μg/ml of Dy488 labeled WT nBT062, stable nBT062, half nBT062 or bispecific nBT062-natalizumab antibodies or natalizumab in PBS/3%FBS. Cells were washed once and incubated in growth media for 3 h at 37°C to enable antibody internalization. Subsequently, extracellular surface anchored CD138 including non-internalized antibodies was removed by Trypsin digest (10 min/37°C).

After washing, cells were fixed in 4% paraformaldehyde and permeabilized by 0.02% Saponin (Sigma-Aldrich). Intracellular blocking was performed in 1x BMB/PBS (Boehringer Blocking Agent, Roche) for 10 min at RT. Cells were washed twice in PBS/3%FBS. Co-staining with lysosomal-associated membrane protein 1 (LAMP1) was performed by polyclonal anti-LAMP1 incubation (Abcam, ab24170, 1:500 in 1x BMB/PBS) followed by two washing steps and staining with a Cy3-labeled goat anti-rabbit H+L secondary antibody (Jackson ImmunoResearch, 111-165-003, 1:500 in 1xBMB/PBS). For sample finalization, cells were washed twice in PBS/3%FBS, resuspended in Prolong Gold Mounting Medium with DAPI (Thermo Fisher Scientific) and transferred onto microscopy slides. Data evaluation was done on the following day using an Olympus IX53 inverted fluorescence microscope (Olympus) and CellSens Software (Olympus).

### DM4 conjugation

To create anti-CD138 ADCs, the conjugation technologie of Immunogen (USA) was used to attach the maytansin derivate DM4 onto WT nBT062, stable nBT062, half nBT062 as well as onto bispecific nBT062-natalizumab. In a first step, the antibody formulation buffer was exchanged by conjugation buffer (50 mM potassium phosphate (Merck), 50 mM sodium chloride (Merck), 2 mM EDTA (Merck), pH 6.5) using a NAP-25 column (GE Healthcare) and protein solutions were concentrated to 10 mg/ml using Amicon Ultra 30 kDa MWCO centrifugation filters (Merck). The N-succinimidyl-4-(2-pyridyldithio)butanoate (SPDB) Linker (Immunogen, solved in 100% Ethanol) was added in a 6.5-times molar quantity to the antibody. Final linker conjugation reaction included an antibody concentration of 8 mg/ml and 5% Ethanol. The reaction was performed for 4 h at RT while stirring. SPDB-modified antibodies were filtered and purified by NAP-25 column using conjugation buffer. Subsequently, DM4 (Immunogen, 15 mM in Dimethylacetamid (DMA, Merck)) was added in a 7.1-times molar quantity to the antibody. DMA was added to a final concentration of 6% and the target antibody concentration was 5 mg/ml. The DM4 conjugation reaction was carried out for 16 h at RT during stirring. A NAP-25 column was used for purification and buffer exchange to antibody formulation buffer followed by a 0.2 μm filtration. The half nBT062 was conjugated with identical amounts of SPDB and DM4 compared to full length antibodies WT nBT062 and stable nBT062 assuming H chain dimerization of this model during the conjugation reaction. To mimic the situation of a shuffled antibody *in vivo*, the bispecific nBT062-natalizumab was conjugated with 50% of SPDB and 50% of DM4.

### DM4-to-antibody ratio and DM4 distribution

The determination of the DM4 to antibody ratio is based on the measurement of the optical density (OD) at 252 and 280 nm. Formulation buffer was used as blank. The DM4 to antibody ratio was calculated using the following formulas:

$$DM4\;[µM]=\frac{(OD252*dilution\;factor)-(0.344*OD280*dilution\;factor)*1000000}{24377}$$

$$BT062\;variant\;[µM]=\frac{\left(1000000*OD280*dilution\;factor)-(5180*DM4\;[µM]\right)}{216060}$$

$$DM4\;to\;antibody\;ratio=\frac{DM4\;[µM]}{BT062\;variant\;[µM]}$$

### *In vitro* cytotoxicity

To investigate the cytotoxicity of the four different DM4-conjugated nBT062 variants, 1x10^4^ CD138-expressing NCI-H929 cells were seeded into 96 well plates on the day before the experiment and were incubated overnight at 37°C. Jurkat cells (CD138^-^/CD49d^+^) were used as negative control cells. WT nBT062-DM4, stable nBT062-DM4, half nBT062-DM4 or bispecific nBT062-natalizumab-DM4 were added to the cells with final antibody-based concentrations of 4, 1, 0.4, 0.2, 0.1, 0.06, 0.04, 0.02, 0.01 and 0 nM. Natalizumab was used as control with 50x excess. Cells were incubated and after 5 days the viability was determined using the WST-1 cell proliferation assay (Roche) according to the manufactures’ instructions. Data evaluation was done using GraphPad Prism 6.1.

### Xenograft mouse study

Animal experiments were performed at Oncotest GmbH (Freiburg, Germany). Experiments were approved by the Committee on the Ethics of Animal Experiments of the Regierungspräsidium Freiburg (Freiburg, Germany; permit number: G-13/13) and conducted according to the guidelines of the German Animal Welfare Act (Tierschutzgesetz). Research staff has relevant education and is FELASA-B qualified. During surgery animals were anesthetized by inhalation of isoflurane.

Human primary MAXF1322 mammary carcinoma (Oncotest) passaged in donor mice was subcutaneously inoculated as 3-4mm fragments into the flank of recipient NMRI nude mice. Animals bearing tumors of 50–250 mm^3^ (preferably 80–200 mm^3^) were randomized to the study groups (5 mice/group). WT nBT062-DM4, stable nBT062-DM4, half nBT062-DM4 and bispecific nBT062-natlizumab-DM4 were administered i.v. as monotherapy either using an antibody-based dose of 4 or 2 mg/kg/week for three injections in total. To investigate whether the efficacy of the model antibodies was influenced by endogenous IgG4, all monotherapy groups received additionally a 10% intravenous immunoglobulin G preparation (IVIg, Intratect 10%, Biotest AG). Intratect 10% was administered i.v. one hour before anti-CD138 antibodies at a dose of 10 ml/kg/week. PBS was used as vehicle control. Mortality was assessed daily; body weight and absolute tumor volume was measured twice-weekly. Tumor volume was calculated as (A x B^2^) x 0.5, where A was the largest and B the perpendicular tumor diameter; tumor volume was calculated relative to day 0 (Start of treatment). The experiment was ended on day 86 after start of treatment or when tumors reached a volume of 2000 mm^3^, whichever came first. According to animal welfare regulations, the following criteria for immediate euthanasia were applied to individual animals, irrespective of the experimental status: (I) tumor volume > 2000 mm^3^; (II) ulcerating, skin-penetrating tumor; (III) body weight loss > 30% on any one measuring day; (IV) recorded, continued body weight loss > 20% for more than two days; (V) recorded, rapid decrease in body weight > 20% within two days; (VI) severe impairment of general condition (apathy, pain, markedly reduced feed and water intake, dyspnea, abnormal habitus or behavior). Animals were euthanized by CO_2_ or cervical dislocation. Efficacy of the ADCs was assessed as median relative tumor volume of the test group compared to the control group. Criteria are shown in Table 1.

**Table 1:** Tumor control efficacy criteria.

Classification	T/C*
Inactive	≥65%
Borderline	50%–<65%
Moderate	25%–<50%
High	10%–<25%
Very high	5%–<10%
Complete remission	<5%

*T/C = median tumor volume in test group/median tumor volume in control group

For graphical demonstration of tumor growth curves, tumor volumes of animals that were terminated due to their tumor load were carried forward using the Last-Obersation-Carried-Forward methodology for as long as this increased the group median tumor volume. To evaluate the differences in time to tumor progression, the cut off of absolute tumor volume was set to 2000 mm^2^ and Kaplan-Meier survival-style statistics combined with the log-rank Mantel-Cox test for pairwise comparisons were employed [30].

## Results

### nBT062 model antibodies are capable of antigen-specific binding

**Fig 2:**
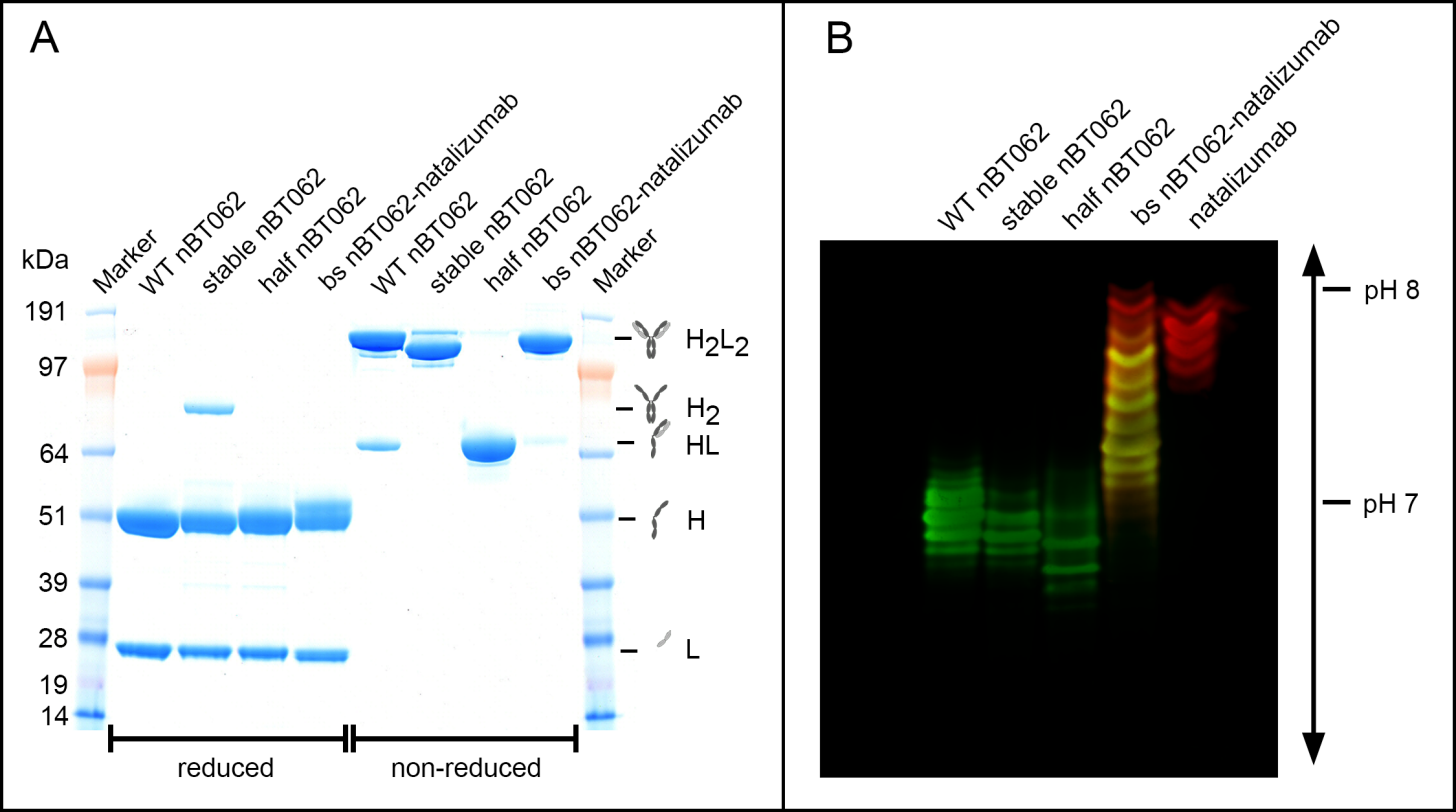
Analytical characterization of nBT062 model variants. (A) Reducing and non-reducing SDS-PAGE analysis of indicated nBT062 variants. L = light chain, H = heavy chain, HL = half antibody, H2 = heavy chain dimer, H2L2 = intact antibody. Molecular weight and antibody explanations are representative for reducing and non-reducing conditions. (B) Indicated nBT062 model variants were separated by isoelectric focusing and blotted onto a PVDF membrane. Selective BT062 and natalizumab specificities were detected by anti-idiotypic antibodies visualized in green and red respectively. The bispecific nBT062-natalizumab fraction is showing two natalizumab exclusive bands.

To study the IgG4-related half-antibody exchange and its potential effect on the antibodies functionality and efficacy, we have generated four anti-CD138 antibodies: (I) WT nBT062, comprises the backbone of an unmodified human IgG4 antibody. (II) Stable nBT062 was generated by introducing stabilizing changes within the hinge and CH3 domains into the WT nBT062 to prevent half antibody exchange. (III) By exchanging the cysteines at position 226 and 229 in the hinge region to serines in WT nBT062, covalent dimerization of the heavy chains was inhibited resulting in nBT062 half antibodies (half nBT062). (IV) To study effects of bispecific antibodies derived by half antibody exchange with endogenous IgG4, a stabilized, bispecific nBT062-natalizumab was designed (Fig 1B). All variants were expressed in FreeStyle CHO-S cells and purified by Protein A affinity chromatography. Reducing and non-reducing SDS-PAGE analysis demonstrated expression of all generated proteins and their expected molecular weights (MW; Fig 2A). While in purified WT nBT062 a small fraction of half antibodies was detectable at approx. 70kDa under non-reducing conditions, no half antibodies were present in stable nBT062 and bispecific nBT062-natalizumab preparations. C226S and C229S mutation in the hinge region of half nBT062 resulted in no full length H2L2 antibodies at approx. 150 kDa. Interestingly, non-reducing, non-denaturing size exclusion chromatography (SEC) revealed dimerization of half nBT062 antibodies assuming their conformation status depends on surrounding conditions (S1 Fig). Reducing SDS-PAGE demonstrated heavy and light chains of all four nBT062 variants at approx. 50 kDa and 25 kDa, respectively. For bispecific nBT062-natalizumab, two heavy chain bands with minimal different molecular weights were observed indicating the separate nBT062 and natalizumab heavy chain. Within the stable nBT062 preparation an additional band at approx. 85 kDa was detected, indicating a H2 fraction. Multicolor western blotting of isoelectric focusing revealed that bispecific nBT062-natalizumab did not contain monospecific nBT062, but a minimal fraction of monospecific natalizumab (Fig 2B). Since this phenomenon is known for the knob and hole engineering technology [31], natalizumab was chosen to comprise the hole-heavy chain to prevent influence on data from monospecific nBT062 within this model. SEC analysis obtained higher amounts of aggregates for stable nBT062 compared to the other variants, which might be an effect of a non-optimized formulation buffer (S1 Fig). Evidence for the prevention of half antibody exchange by S228P and R409K mutations introduced into stable nBT062 and bispecific nBT062-natalizumab was demonstrated by an *in vitro* assay: Exposing stable nBT062 and WT nBT062 in the presence of natalizumab with 10 mM reduced glutathione and subsequent re-oxidation with oxidized glutathione resulted in fraction of bispecific WT nBT062/natalizumab antibodies, while such antibody shuffling was not observed for stable nBT062 (S2 Fig).

**Fig 3:**
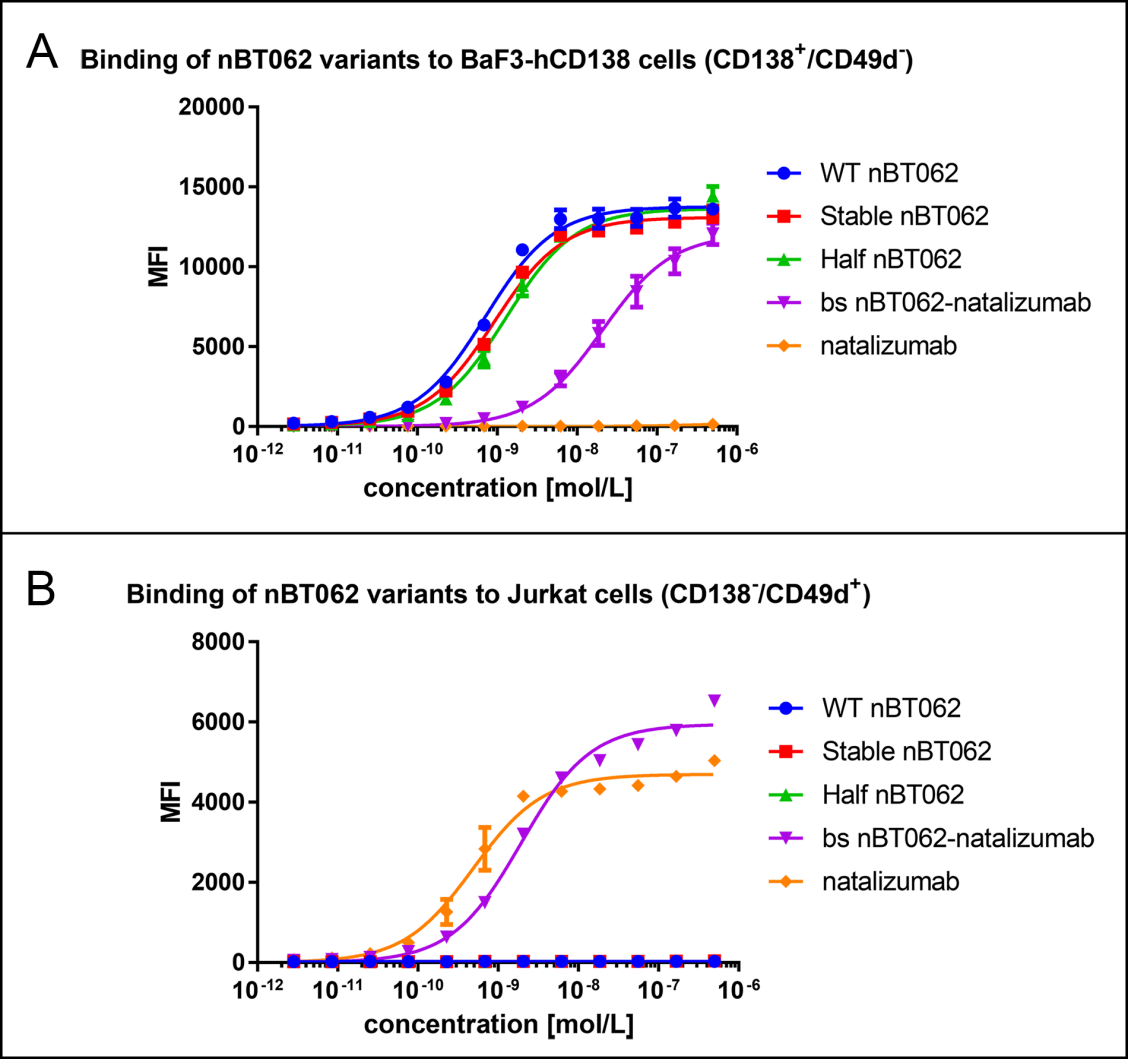
nBT062 model variants selectively bind to CD138^+^ cells in a dose-depended manner. BaF3-hCD138 cells, positive for nBT062 antigen and negative for natalizumab antigen (A), and Jurkat cells, negative for nBT062 antigen and positive for natalizumab antigen (B), were incubated with different concentrations (5x10^-7^–2.82x10^-12^ M) of WT nBT062, stable nBT062, half nBT062 and bispecific nBT062-natalizumab antibodies. Data was obtained via flow cytometry using a secondary anti-human antibody for detection. Binding curves demonstrate one representative experiment out of three, each measured in triplicates. MFI = Median Fluorescence Intensity.

Flow cytometric analysis was used to compare specific antigen binding of the 4 different nBT062 antibody variants (Fig 3, Table 2). While WT nBT062, stable nBT062 and half nBT062 antibodies showed similar binding to BaF3-hCD138 (CD138^+^/CD49d^-^) cells, the binding of bispecific nBT062-natalizumab was slightly reduced. However, the affinity of nBT062-natalizumab against CD138 was still in the nanomolar range (Table 2). Additionally, the bispecific nBT062-natalizumab antibody exhibited binding of CD49d-expressing, CD138^-^ Jurkat cells similar to natalizumab (Fig 3B), indicating that both antigens were recognized by this model antibody.

**Table 2:** Antibody binding affinities towards BaF3-hCD138 (CD138^+^/CD49d^-^) and Jurkat (CD138^-^/CD49d^+^) cells calculated from binding curves of Fig 3.

Cells	WT nBT062 [M]	Stable nBT062 [M]	Half nBT062 [M]	Bs nBT062-natalizumab [M]	natalizumab [M]
**BaF3-hCD138**	7.13x10-10	9.17x10-10	1.30x10-9	2.08x10-8	n/a
**Jurkat**	n/a	n/a	n/a	1.93x10-9	4.90x10-10

### All nBT062 model antibodies are internalized upon CD138-specific binding

CD138-mediated antibody uptake into CD138^+^ cells was investigated by flow cytometry and fluorescence microscopy.

**Fig 4:**
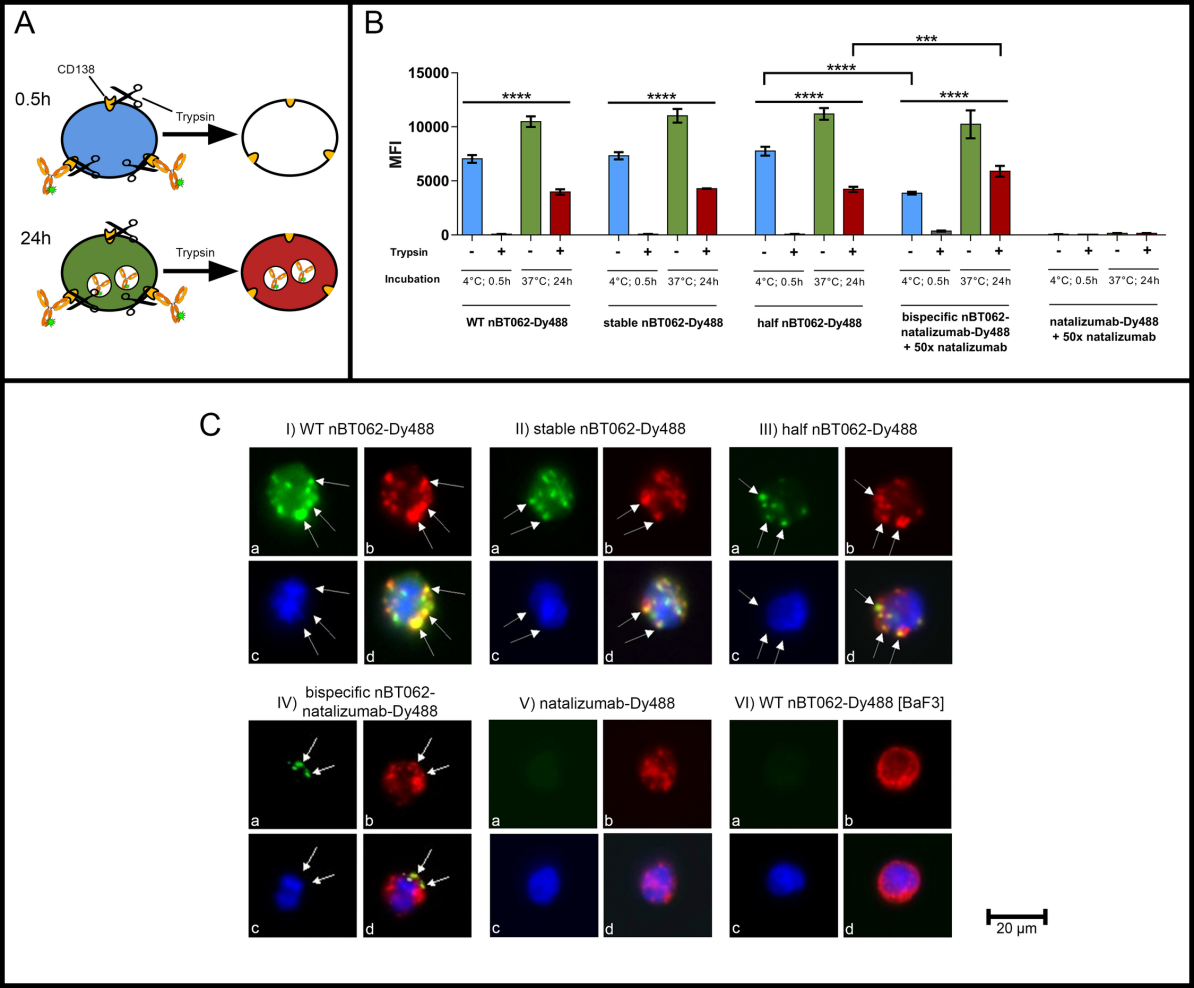
All nBT062 model variants are internalized *in vitro*. (A) Schematic overview of measured binding/internalization status of fluorescent labeled antibodies. (B) Flow cytometric internalization analysis of WT nBT062, stable nBT062, half nBT062 and bispecific nBT062-natalizumab. CD49d was blocked by 50x excess of unlabeled natalizumab. Data represent median fluorescence intensity values of at least three separate experiments. Column colors represent antibody location as shown in (A). ****p<0.0001, ***p<0.001. (C) Fluorescence microscopy of BaF3-hCD138 (CD138^+^) cells (I-V) or BaF3 (CD138^-^) control cells (VI) incubated for 3h with indicated Dylight-448 labeled nBT062 model antibodies or natalizumab (green, a). Lysosomal-associated membrane protein-1 (LAMP-1) is shown in red (b), nucleus is shown in blue (c). Respective overlay pictures are demonstrated (d).

For flow cytometric analysis Dy488 labeled nBT062 variants were added to CD138^+^ NCI-H929 cells for either 0.5 h or 24 h. To distinguish between surface-bound and internalized antibodies, cells were treated with or without Trypsin before the measurement (Fig 4A and 4B). Antibody internalization patterns including initial surface binding and internalized antibodies were comparable for WT nBT062, stable nBT062 and half nBT062. Despite the excess of each nBT062 variant during the 24 h incubation, the detected internalized antibody levels were approx. half of the initial bound antibody levels. In contrast to the monospecific variants, bispecific nBT062-natalizumab demonstrated a reduced initial surface staining but a significantly higher fluorescence intensity of internalized antibodies. Binding and potential internalization of bispecific nBT062-natalizumab due to CD49d was successfully blocked by an excess of natalizumab. One possible explanation might be the lack of CD138 cross-linking potentially increasing the internalization process itself. This might take place under the premise of keeping in mind that the half nBT062 model still possesses the potential to dimerize due to non-covalent interactions between the CH3 domains of two heavy chains.

To further investigate the type of internalization, epifluorescence microscopy was performed after 3 hours of cell incubation with the four different fluorescent-labeled nBT062 antibodies. Co-localization with an antibody targeting the Lysosomal-associated membrane protein 1 (LAMP-1) indicated a CD138-mediated uptake by the endosomal/ lysosomal pathway (Fig 4C).

### The DM4-conjugation is not influenced by mutations and does not interfere with antigen binding

To generate antigen-specific cytotoxic variants of the different nBT062 models, they were conjugated to DM4. The striven drug-to-antibody ratio for each model variant was based on clinical BT062 material and respective to the expected ratio for mimicking the *in vivo* situation, e.g. two half nBT062-DM4 molecules should contain the same amount of toxin compared to one WT nBT062-DM4 or stable nBT062-DM4. In addition, the bispecific nBT062-natalizumab was linked with 50% of the DM4 amount to mimic a potential half-antibody exchange of BT062 with endogenous IgG4 species. It was observed that the conjugation reaction was proportional in terms of the used amount of SPDB linker and DM4. No influence on the conjugation reaction was found due to the introduced mutations of each model variant. Results of measured DM4-to-antibody ratios are shown in Table 3.

**Table 3:** DM4-to-antibody ratios of wild type (WT), stable and half nBT062 and bispecific nBT062-natalizumab.

Antibody model	DM4-to-antibody ratio
WT nBT062-DM4	3.5
Stable nBT062-DM4	3.4
Dimerized (2x)half nBT062-DM4	3.2
Bispecific nBT062-natalizumab-DM4	1.7

FACS analysis was used to evaluate the influence of DM4-conjugation on the binding activity of the different nBT062 model variants. However, no significant difference in binding between conjugated or unconjugated nBT062 variants to CD138^+^ cells was observed. Binding was in the low nanomolar range as shown in Table 4.

**Table 4:** Binding avidity of unconjugated or DM4-conjugated WT nBT062, stable nBT062, half nBT062 and bispecific nBT062-natalizumab to CD138-positive cells measured by flow cytometry in three independent experiments.

Cells	DM4-conjugate	WT nBT062 [M]	Stable nBT062 [M]	Half nBT062 [M]	bispecific nBT062-natalizumab [M]
NCI-H929	No	1.16E-09	1.57E-09	1.88E-09	9.25E-09*
NCI-H929	Yes	1.68E-09	1.79E-09	2.02E-09	7.89E-09*
BaF3-hCD138	No	5.46E-10	7.05E-10	1.12E-09	2.26E-08
BaF3-hCD138	Yes	7.28E-10	7.00E-10	9.00E-10	1.79E-08

*Bispecific nBT062-natalizumab is capable of CD49d binding on NCI-H929 cells.

### nBT062-DM4 model antibodies mediate CD138-specific cytotoxicity *in vitro*

**Fig 5:**
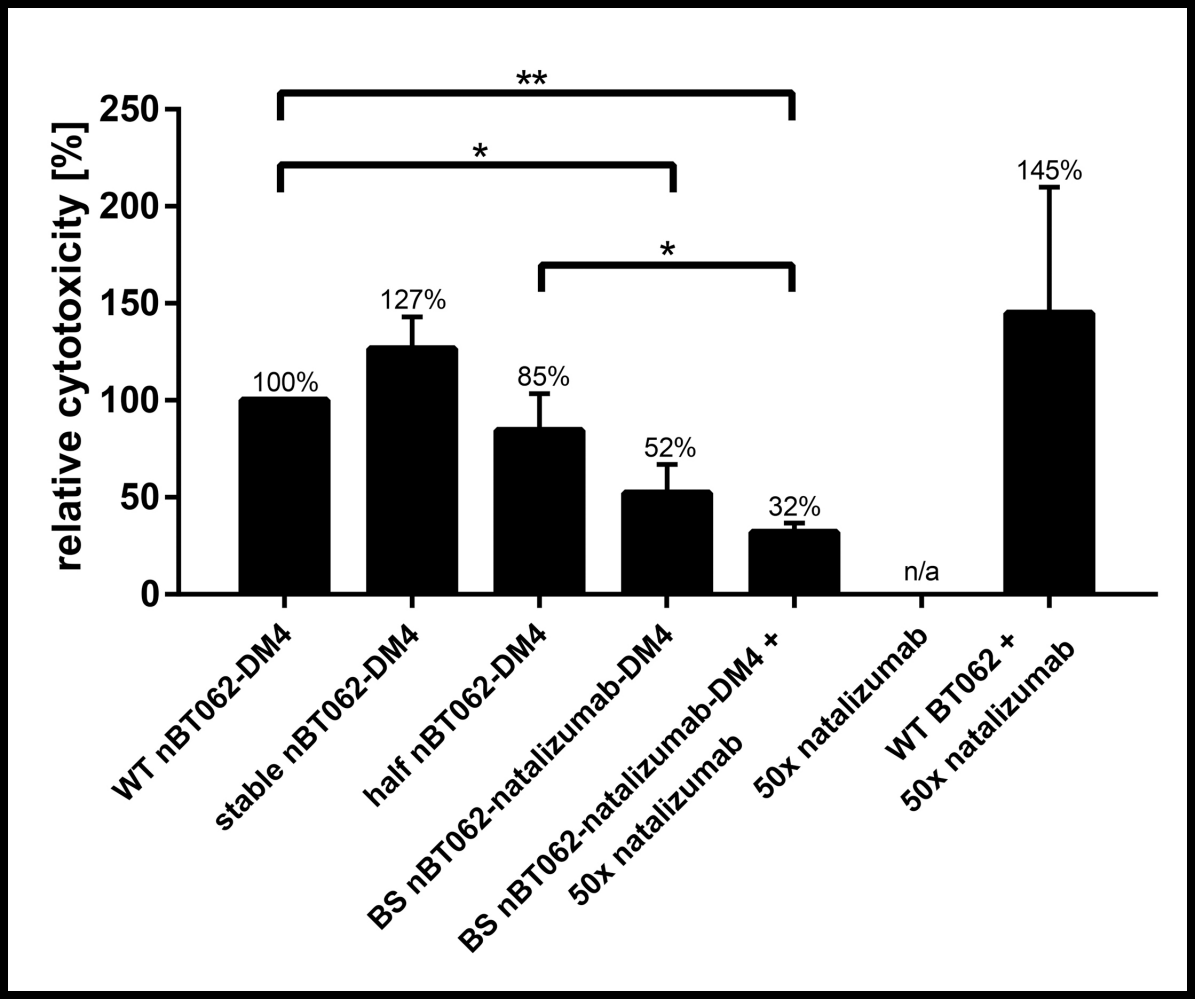
nBT062-DM4 model variants kill CD138^+^ NCI-H929 tumor cells *in vitro*. NCI-H929 cells (CD138^+^/CD49d^+^) were incubated for 5 days with varying concentrations of WT nBT062-DM4, stable nBT062-DM4, half nBT062-DM4 and bispecific nBT062-natalizumab-DM4 antibodies. Natalizumab was used for CD49d blocking. Reciprocal IC_50_ values of each antibody-drug conjugate were normalized to WT nBT062-DM4 and one DM4 molecule per antibody. Results are shown from three separate experiments each measured in triplicates. *p<0.05, **p<0.01.

The selective cytotoxic activity of WT nBT062-DM4, stable nBT062-DM4, half nBT062-DM4 and bispecific nBT062-natalizumab-DM4 was investigated using a cytotoxicity assay towards NCI-H929 (CD138^+^/ CD49d^+^) multiple myeloma cells. Based on three individual experiments each measured in triplicates, WT nBT062-DM4, stable nBT062-DM4 and half nBT062-DM4 showed similar half maximal inhibitory antibody-based concentrations (IC_50_) of 0.105 ± 0.019, 0.085 ± 0.005, and 0.137 ± 0.006 nM, respectively. On the contrary, bispecific nBT062-natalizumab-DM4 demonstrated a decreased IC_50_ of 0.456 ± 0.228 nM (S3A Fig). Additional data processing revealed that bispecific nBT062-natalizumab-DM4 attained 52% of WT nBT062-DM4 cytotoxicity based on a DM4-to-antibody-normalized evaluation (Fig 5). To additionally prevent bispecific nBT062-natalizumab-DM4 antibodies from binding to CD49d on NCI-H929 cells, natalizumab was co-incubated with a 50x excess. While natalizumab itself did not mediate any cytotoxic effect, there was an insignificant trend to decrease the efficacy of bispecific BT062-natalizumab-DM4. However, WT nBT062 was also slightly influenced by natalizumab in terms of demonstrating a higher variability (Fig 5). CD138^-^ Jurkat cells were not affected by nBT062-DM4 variants in concentrations tested in this assay (S3B Fig). Taken together, we were able to demonstrate that bispecific nBT062-natalizumab-DM4 antibodies were still able to induce CD138-mediated cytotoxicity at low nanomolar concentrations.

### Stabilizing mutations improve the *in vivo* efficacy of nBT062 variants in the presence of human IgG

In addition to *in vitro* testing, we investigated if nBT062-DM4 and potentially derived antibodies species thereof, mimicked by the different model variants, were still capable of specific tumor cell killing *in vivo*. NMRI nude mice bearing subcutaneous xenografts of the patient-derived mammary cancer MAXF 1322 were treated three times with either 4 or 2 mg/kg/week of WT nBT062-DM4, stable nBT062-DM4, half nBT062-DM4 and bispecific nBT062-natalizumab-DM4. To determine the influence of endogenous IgG4 antibodies on the therapeutic antibodies’ efficacy, nBT062-DM4 model variants were administered either alone or in combination with 10 ml/kg/week of a 10% IVIg preparation containing also the IgG4 subtype. The injection of the IVIg resulted in a serum concentration comparable to human IgG4 serum levels ranging from 10 µg/ml to > 2 mg/ml [32].

**Fig 6:**
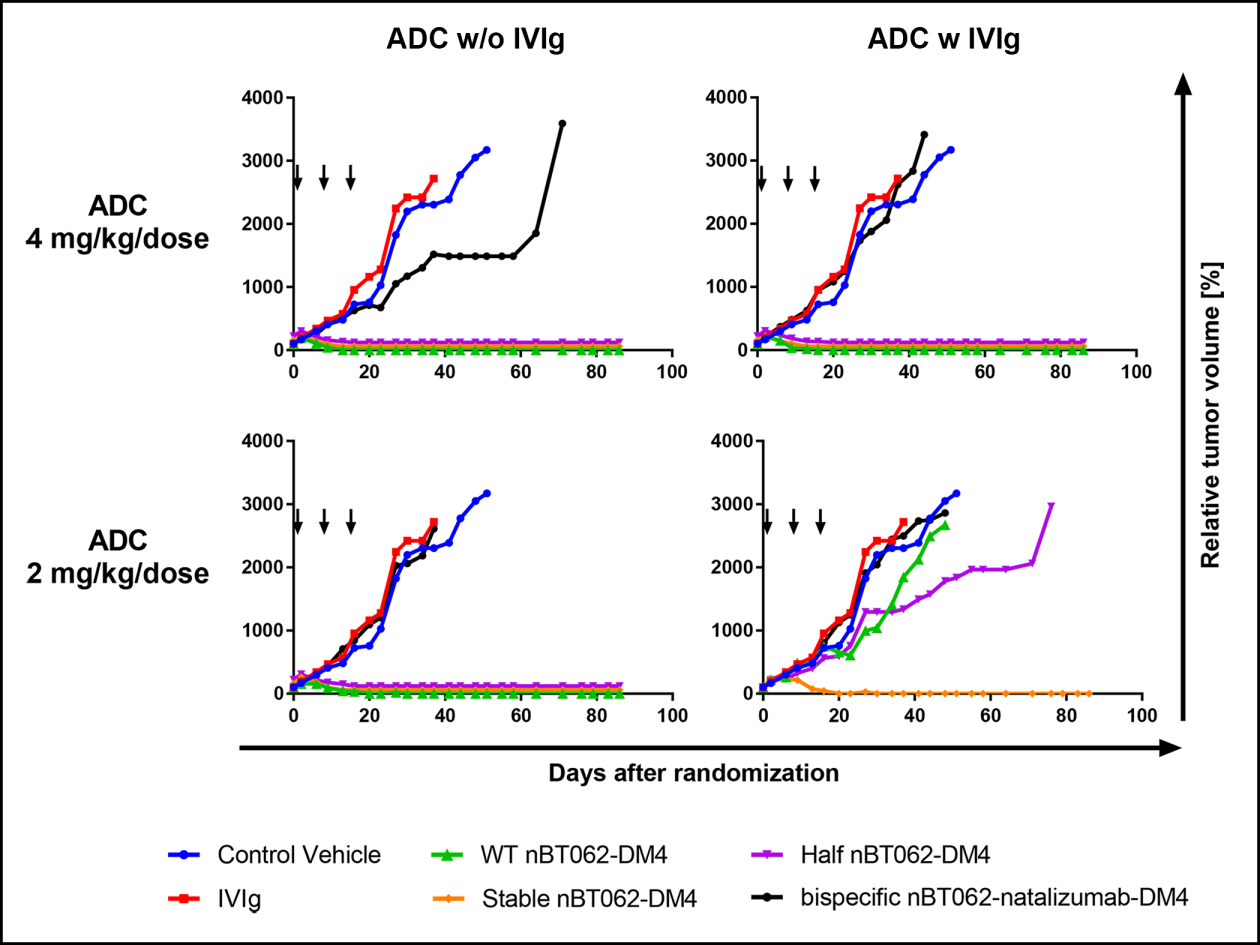
Half-antibody exchange preventing mutations of nBT062-DM4 models improve the tumor growth inhibition in the presence of human IgG against CD138^+^ human mammary carcinoma xenografts in NMRI nude mice. Mice bearing established xenograft tumors were treated with three once weekly i.v. injections (indicated by arrows) of WT nBT062-DM4, stable nBT062-DM4, half nBT062-DM4 and bispecific nBT062-natalizumab-DM4 either alone or in combination with a 10% intravenous immunoglobulin G (IVIg) preparation administered i.v. 10 ml/kg/week. PBS was used as vehicle control and IVIg was also tested as monotherapy. The nBT062-DM4 model variants were tested using doses of 4 mg/kg/week or 2 mg/kg/week.

**Fig 7:**
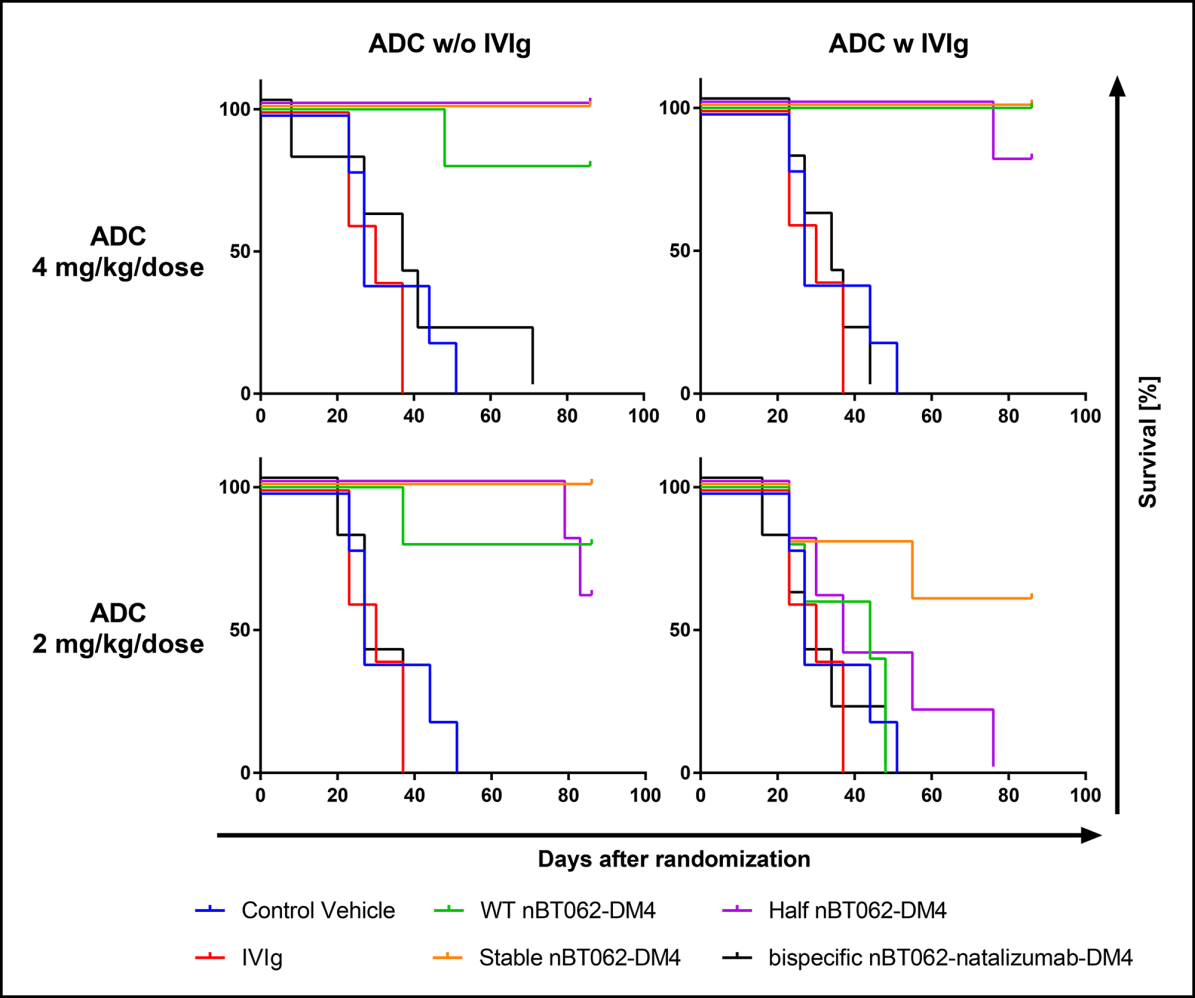
Half-antibody exchange preventing mutations of nBT062-DM4 models improve survival in the presence of human IgG against CD138+ human mammary carcinoma xenografts in NMRI nude mice. Survival curves respective to tumor growth curves from Fig 6 are shown. Mice bearing established xenograft tumors were treated with three once weekly i.v. injections of WT nBT062-DM4, stable nBT062-DM4, half nBT062-DM4 and bispecific nBT062-natalizumab-DM4 either alone or in combination with a 10% intravenous immunoglobulin G (IVIg) preparation administered i.v. 10 ml/kg/week. PBS was used as vehicle control and IVIg was also tested as monotherapy. The nBT062-DM4 model variants were tested using doses of 4 mg/kg/week or 2 mg/kg/week.

Tumor growth curves are shown in Fig 6 and Kaplan-Meier survival curves are depictured in Fig 7. 4 mg/kg/week injections of WT nBT062-DM4, stable nBT062-DM4 and half nBT062-DM4 induced complete tumor remission in treated animals (min. T/C: 0.0%) and significant survival compared to vehicle control (S1 Table). Even after the treatment phase of 21 days no tumor regrowth was detected until observation on day 86 (data not shown). Co-administration of the human IgG preparation had no influence on the treatment outcome (Mantel-Cox test: p>0.3173). Overall the bispecific nBT062-natalizumab-DM4 at 4 mg/kg/week alone demonstrated a borderline efficacy (min. T/C: 62.1%), while one treated animal within this group showed a very good response (opt. T/C: 8.2). However, survival was not significantly improved by bispecific nBT062-natalizumab-DM4 compared to vehicle control. The antitumor effect was strongly decreased in the presence of human IgGs. Direct blocking of CD138 by the IgG preparation was excluded, since no binding to human CD138 was detected by ELISA (data not shown). At reduced doses of 2 mg/kg/week WT nBT062-DM4, stable nBT062-DM4 and half nBT062-DM4 complete remissions without tumor regrowth after the treatment phase were seen in most treated animals (min. T/C: 0.0%) resulting in significant prolongation of survival compared to vehicle control and IVIg alone (p<0.0132).

Interestingly, co-administration of human IgG decreased the observed antitumor effects: Half nBT062-DM4 showed a moderate efficacy (min. T/C: 45.3%) and WT nBT062-DM4 exhibited only a borderline efficacy (min. T/C: 58.9). In both cases the decrease was statistically significant based on the Mantel-Cox analysis on survival curves (p<0.0251). However, the antitumor effect of stable nBT062-DM4 was only slightly, but not significantly affected by human IgGs: Complete remission and survival was seen in 3 of 5 mice (min. T/C: 0.0%; Fig 7). Bispecific nBT062-natalizumab-DM4 was inactive at this dose. These data demonstrate that nBT062-DM4 and the models mimicking potentially related IgG4 species are all capable of CD138^+^ tumor cell killing *in vivo*. Nevertheless, the reduced efficacy of WT nBT062-DM4 and half nBT062-DM4 in the presence of human IgGs–which was hardly observed for the stable nBT062-DM4 –indicate an IgG4 shuffling attenuated anti-tumor effect. In case of WT nBT062-DM4, this IgG4 shuffling might involve covalent interactions of disulfide bonds between WT nBT062 and any half-antibody of the human IgG4 pool. In contrast, shuffling interactions of half nBT062 with human exogenous half antibodies are based on non-covalent heavy chain dimerizations similar as observed by SEC (S1 Fig).

## Discussion

CD138 is over-expressed in multiple myeloma and on several solid tumors. It is a highly attractive target for cancer therapy [11,16,17]. BT062 (indatuximab ravtansine), an anti-CD138 IgG4 antibody with a wild type hinge region and conjugated to the tubulin inhibiting agent DM4 was able to demonstrate anti-tumor efficacies for multiple myeloma in preclinical experiments [18,33]. As recently demonstrated, endogenous and unstabilized therapeutic IgG4 antibodies undergo a dynamic process *in vivo* called antibody shuffling. During this process, they are able to exchange half antibodies with each other. Thereby bispecific antibodies acting functionally monovalent for each antigen are created [4,24,34]. In this study we have generated BT062 model antibodies mimicking potential *in vivo* derived BT062 species to investigate their functionality in terms of tumor cell killing *in vitro* and *in vivo*. We demonstrated that all antibody species (WT nBT062, stable nBT062, half nBT062 and bispecific nBT062-natalizumab) were capable of CD138 binding, internalization and, when conjugated to DM4, tumor cell killing *in vitro* and *in vivo*. While WT nBT062(-DM4), stable nBT062(-DM4) and half nBT062(-DM4) showed very similar functional properties in *in vitro* assays, the bispecific nBT062-natalizumab(-DM4) displayed reduced binding and CD138-specific cytotoxicity. This decreased binding activity might be due to the lack of bivalent antigen attachment, while the diminished cytotoxicity might be mainly a result of payload reduction in terms of DM4 cell transport limitations. Nevertheless, the IC_50_ was still below <10^−9^ M which is highly effective compared to other multiple myeloma drugs [35,36].

In xenograft studies the presence of human IgGs had only a minor influence on the antitumor efficacy of stable nBT062-DM4, while the efficacy of WT nBT062-DM4 and half nBT062-DM4 was reduced. Half nBT062-DM4 antibodies were only capable of non-covalent heavy chain pairing. Thus, the results suggest that IgG4 shuffling occurred in both unstabilzed ADC models, WT nBT062-DM4 and half nBT062-DM4. This is in line with data from Labrijn et al. demonstrating that S228P and R409K mutations separately lead to the prevention of IgG4 shuffling [7]. Bispecific nBT062-natalizumab-DM4 antibodies were only effective at the higher tested dose without the addition of IgG antibodies. Interestingly, there was an trend that the efficacy of stable nBT062-DM4 and bispecific nBT062-natalizumab-DM4 both harboring mutations preventing IgG4 shuffling was slightly influenced by co-administration of a human IgG preparation. No anti-CD138 antibodies were found within the IgG preparation which might have competed with the ADCs. However, a possible explanation might be given based on results of Bleeker et al who demonstrated that clearance of monoclonal IgG antibodies in mice was increased by the injection of IVIgs [37]. As immunodeficient mice do not contain endogenous antibodies, administered monoclonal antibodies or ADCs can bind unhinderedly to the neonatal FcγR (FcRn), a receptor mainly defining the IgG's half-life during circulation. Co-administration of an IVIg preparation results in competed FcRn binding potentially decreasing the half-life of monoclonal antibodies or ADCs. Since WT nBT062-DM4 was much more effective than bispecific nBT062-natalizumab-DM4, it can be assumed that half antibody exchange occurred over time and CD138 receptors on the tumor cells were saturated with monospecific nBT062-DM4 first. This is in accordance with results from fluorescence microscopy demonstrating internalized nBT062 variant antibodies after 3h of incubation. In addition, it is supported by a study of Debaene et al.: They showed that an equilibrium between bispecific and monospecific natalizumab and Hz6F4-2v3 was reached after 24 h of incubation in 0.5 mM glutathione [38].

In terms of ADC creation or therapeutic antigen blocking, IgG4 antibodies are of high interest because they do not mediate effector functions like ADCC and CDC and thus do not directly prime the immune system towards a response against antigen-expressing cells. The influence of half antibody exchange on the efficacy and safety of a therapeutic IgG4 antibody is mainly dependent on its specific mode of action. The development of 1D09C3 for the treatment of advanced B cell malignancies was discontinued, since the loss of bivalent binding rendered the drug inactive for crosslinking human MHCII [39–41]. On the contrary, natalizumab is approved for the treatment of multiples sclerosis and Crohn's disease based on good clinical efficacies and acceptable tolerability, although *in vivo* half antibody exchange was obtained [24,40,42].

As investigated in this study, the efficacy of the hinge-unmodified, therapeutic drug BT062 might be decreased at lower doses by half antibody exchange in patients. This is probably based on a reduced DM4 transport into the tumor cells. The issue on the safety of an ADC able to exchange its half antibodies might be very low for the following reasons: Endogenous IgG4 antibodies might not be associated to endogenous proteins relating to the hypotheses of self/ non-self discrimination of the immune system, but they might be associated to allergens for damping down inflammatory responses as they lack effector functions [43,44]. In addition, the specificities of endogenous IgG4 antibodies are highly variable and thus bispecific ADC-endogenous IgG4 antibodies would probably not generate cytotoxic amounts against an antigen different to the therapeutic target. These assumptions are highly supported by an ongoing clinical trial testing hinge-unmodified BT062 for the treatment of multi myeloma in combination with Dexamethasone and Lenalidomide or Pomalidomide whereas BT062 is well tolerated and showing encouraging activity [20]. This activity seems to be a result of an optimized dosing schedule counteracting a half antibody exchange-based efficacy loss similar as observed by high dose administration in the *in vivo* experiment of this preclinical study.

Taken together, this study demonstrates in preclinical experiments that half antibody exchange reduces the efficacy of antibody-drug conjugates. Thus, the introduction of stabilizing mutations preventing this half antibody exchange is recommended when using IgG4 antibodies as therapeutics.

## Supporting information

S1 Fig Size exclusion chromatography (SEC) of nBT062 model variants. WT nBT062, stable nBT062, half nBT062 and bispecific nBT062-natalizumab were separated under non-denaturing, non-reducing conditions by SEC using a TSK-Gel G3000 column (Tosoh Bioscience) connected to a nano HPLC system (Ultimate 3000, Thermo Fisher Scientific). Detection was done at 280 nm and different antibody-related species are indicated. Aggregates were observed in proportions of 4.5, 7.4, 7.1, and 25% for half nBT062, WT nBT062, bispecific nBT062-natalizumab and stable nBT062, respectively. The retention time’s peak maximum of half nBT052 was minimally reduced compared to the other models, especially in comparison to WT nBT062 and stable nBT062, indicating non-covalent dimerization of heavy chains under the applied conditions. H = Heavy chain, L = light chain.
(TIF)

S2 Fig *In vitro* evaluation of half antibody preventing mutations. WT nBT062 and stable nBT062 were mixed 1:1 with natalizumab (2mg/ml per antibody) and incubated over night at 37°C in the presence of 10 mM reduced glutathione (GSH). GSH was removed by 2x 4 h dialysis against PBS and re-oxidation was performed by incubating the antibody solutions in 5 mM oxidized glutathione (GSSG) over night at 37°C. Re-oxidized mixtures of WT nBT062 + natalizumab and stable nBT062 + natalizumab as well as individual control antibodies (10 µg of each antibody) were separated by isoelectric focusing followed by Coomassie Brilliant Blue staining. Formation of bispecific antibodies was observed for WT nBT062 (red line) while stabilizing mutations S228P and R409K incorporated into stable nBT062 prevent in vitro half antibody exchange under the applied conditions.
(TIF)

S3 Fig *In vitro* cytotoxicity curves of nBT062-DM4 variants. NCI-H929 (CD138+/CD49d-) or Jurkat (CD138-/CD49d+) cells were incubated for five days with different concentrations (4, 1, 0.4, 0.2, 0.1, 0.06, 0.04, 0.02, 0.01 and 0 nM, antibody-based) of WT nBT062-DM4, stable nBT062-DM4, half nBT062-DM4 or bispecific nBT062-natalizumab-DM4 as indicated. CD49d was blocked by 50x excess of natalizumab. WST-1 cell proliferation agent (Roche) was used to determine the fraction of viable cells according to the manufacturer’s instructions. A: Example of one experiment on NCI-H929 cells, each data point was measured in triplicates. Shown are non-linear fitted inhibitory curves. *p<0.05. B: Example of one experiment on Jurkat cells, each data point was measured in triplicates. No ADC dependent cytotoxicity was observed using the applied concentrations.
(TIF)

S1 Table Log-rank (Mantel-Cox) statistical analysis on xenograft mice survival curves. (PDF)

S2 Table Numbers of animals used, euthanized, and the cause of death for all animals. (PDF)

S1 File Minimal data set. (PDF)
