# Supplementary figures and images for: Functional relevance of in vivo half antibody exchange of an IgG4 therapeutic antibody-drug conjugate
Source: PLoS One. 2018 Apr 19;13(4):e0195823. doi: 10.1371/journal.pone.0195823 (PMC5908158; doi:10.1371/journal.pone.0195823)

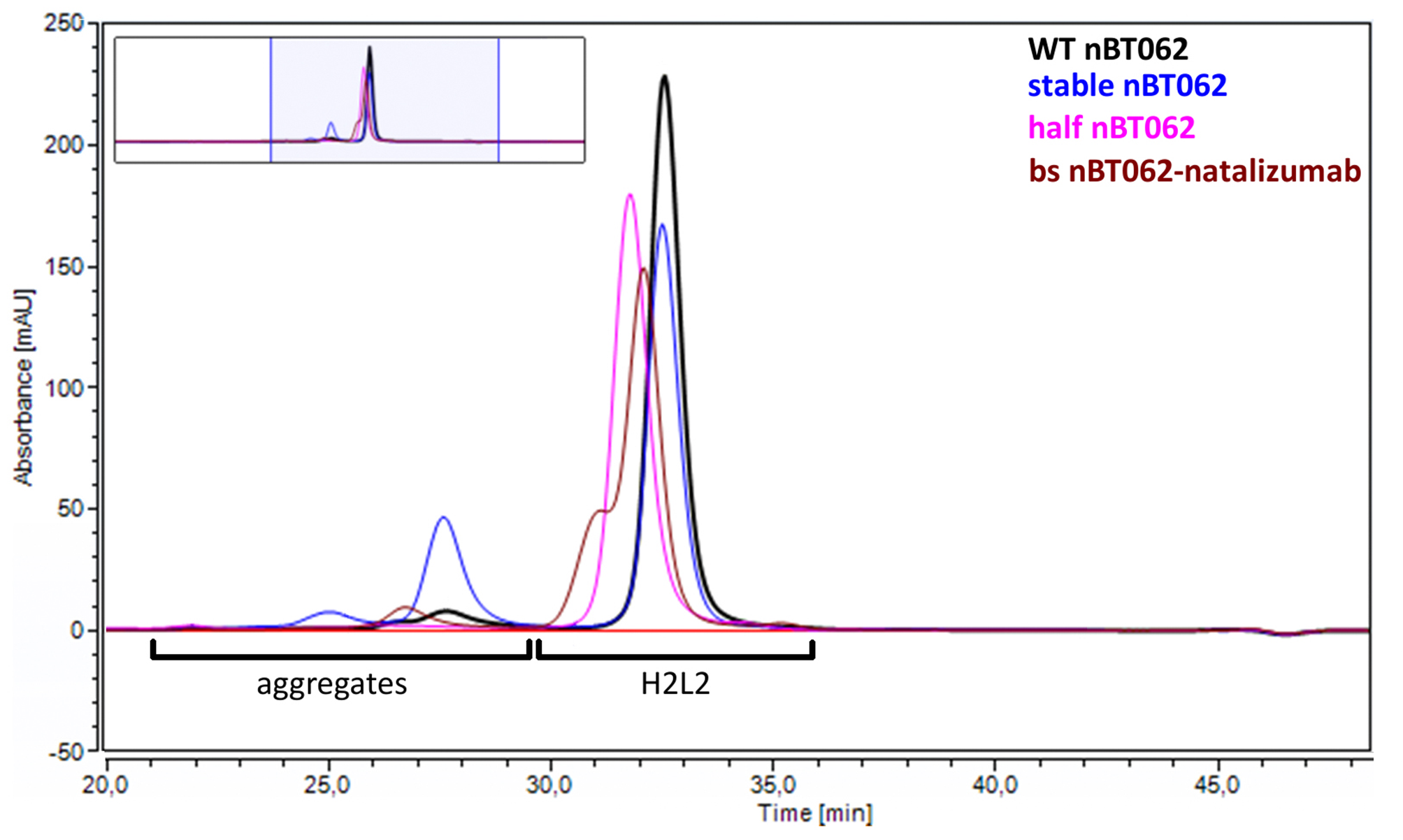

Supplement: S1 Fig — WT nBT062, stable nBT062, half nBT062 and bispecific nBT062-natalizumab were separated under non-denaturing, non-reducing conditions by SEC using a TSK-Gel G3000 column (Tosoh Bioscience) connected to a nano HPLC system (Ultimate 3000, Thermo Fisher Scientific). Detection was done at 280 nm and different antibody-related species are indicated. Aggregates were observed in proportions of 4.5, 7.4, 7.1, and 25% for half nBT062, WT nBT062, bispecific nBT062-natalizumab and stable nBT062, respectively. The retention time’s peak maximum of half nBT052 was minimally reduced compared to the other models, especially in comparison to WT nBT062 and stable nBT062, indicating non-covalent dimerization of heavy chains under the applied conditions. H = Heavy chain, L = light chain. (TIF) [file pone.0195823.s001.tif]

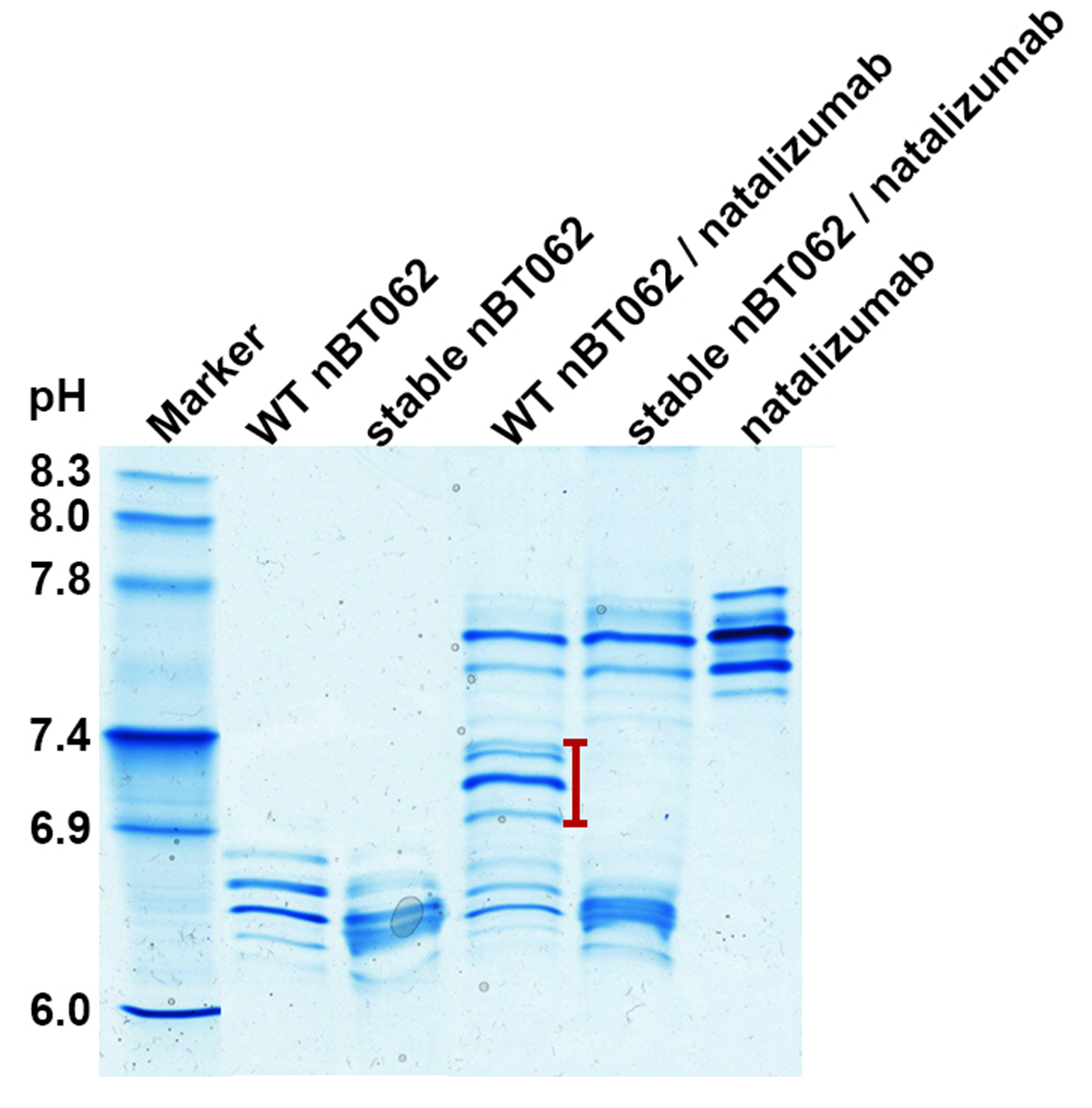

Supplement: S2 Fig — WT nBT062 and stable nBT062 were mixed 1:1 with natalizumab (2mg/ml per antibody) and incubated over night at 37°C in the presence of 10 mM reduced glutathione (GSH). GSH was removed by 2x 4 h dialysis against PBS and re-oxidation was performed by incubating the antibody solutions in 5 mM oxidized glutathione (GSSG) over night at 37°C. Re-oxidized mixtures of WT nBT062 + natalizumab and stable nBT062 + natalizumab as well as individual control antibodies (10 µg of each antibody) were separated by isoelectric focusing followed by Coomassie Brilliant Blue staining. Formation of bispecific antibodies was observed for WT nBT062 (red line) while stabilizing mutations S228P and R409K incorporated into stable nBT062 prevent in vitro half antibody exchange under the applied conditions. (TIF) [file pone.0195823.s002.tif]

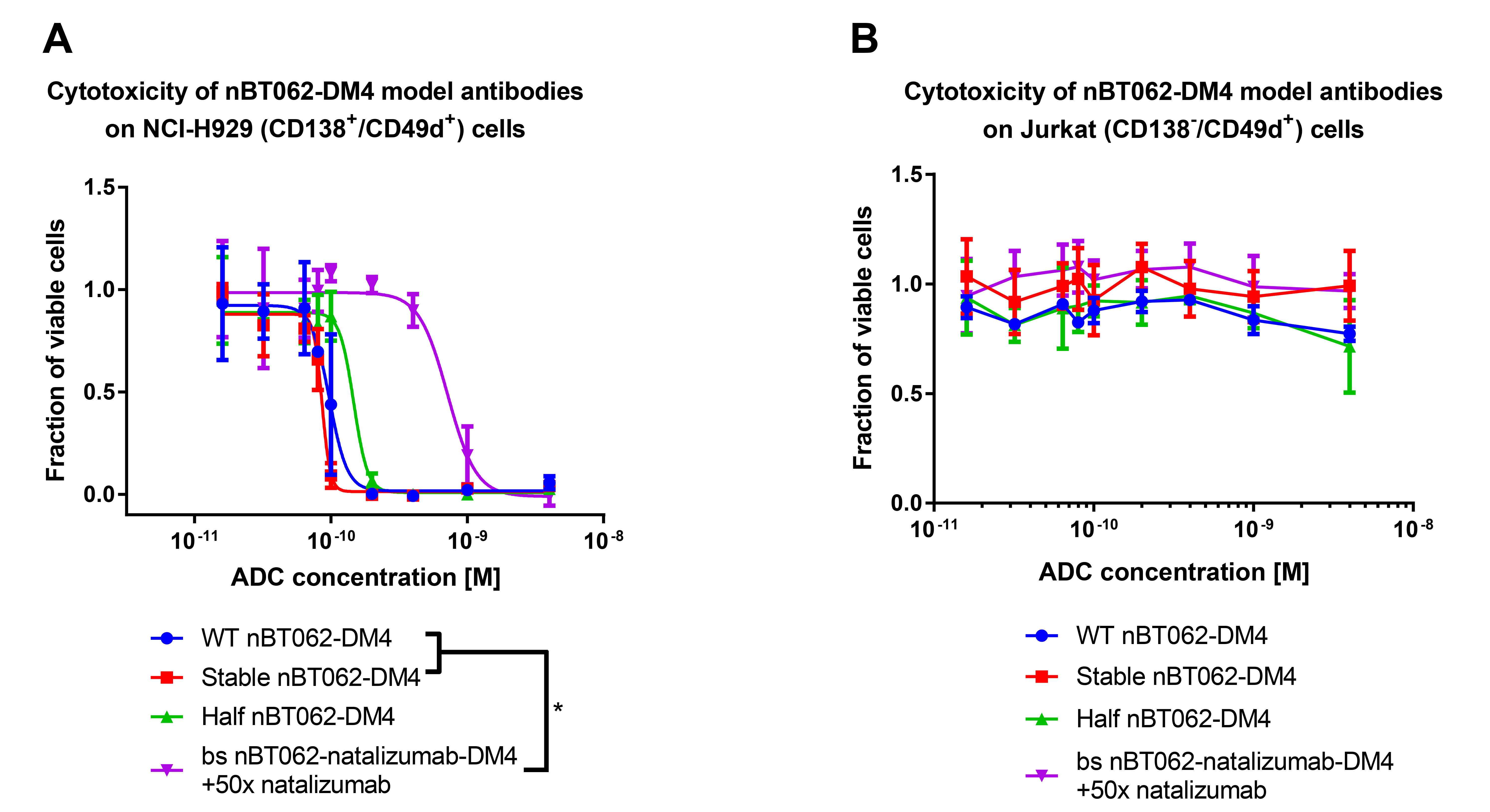

Supplement: S3 Fig — NCI-H929 (CD138+/CD49d-) or Jurkat (CD138-/CD49d+) cells were incubated for five days with different concentrations (4, 1, 0.4, 0.2, 0.1, 0.06, 0.04, 0.02, 0.01 and 0 nM, antibody-based) of WT nBT062-DM4, stable nBT062-DM4, half nBT062-DM4 or bispecific nBT062-natalizumab-DM4 as indicated. CD49d was blocked by 50x excess of natalizumab. WST-1 cell proliferation agent (Roche) was used to determine the fraction of viable cells according to the manufacturer’s instructions. A: Example of one experiment on NCI-H929 cells, each data point was measured in triplicates. Shown are non-linear fitted inhibitory curves. *p<0.05. B: Example of one experiment on Jurkat cells, each data point was measured in triplicates. No ADC dependent cytotoxicity was observed using the applied concentrations. (TIF) [file pone.0195823.s003.tif]
