# Supplementary material for: Functional relevance of in vivo half antibody exchange of an IgG4 therapeutic antibody-drug conjugate
Source: PLoS One. 2018 Apr 19;13(4):e0195823. doi: 10.1371/journal.pone.0195823 (PMC5908158; doi:10.1371/journal.pone.0195823)
Supplement: S1 Table — (PDF) [file pone.0195823.s004.pdf]

S1 Table. Log-rank (Mantel-Cox) statistical analysis on xenograft mice survival curves.

Overall Comparison of Survival Curves
Log-rank (Mantel-Cox) Test

Chi square	89.46
df	17
P value	< 0.0001
P value summary	***
Are the survival curves sig different?	Yes

Mantel-Cox Test of individual groups	Vehicle control	IVIg	WT nBT062 4 mg/kg	stable nBT062 4mg/kg	half nBT062 4 mg/kg	bs nBT062-natalizumab 4 mg/kg	WT nBT062 4 mg/kg + IVIg	stable nBT062 4 mg/kg + IVIg	half nBT062 4 mg/kg + IVIg	bs nBT062-natalizumab 4mg/kg + IVIg	WT nBT062 2 mg/kg	stable nBT062 2 mg/kg	half nBT062 2 mg/kg	bs nBT062-natalizumab 2 mg/kg	WT nBT062 2 mg/kg + IVIg	stable nBT062 2 mg/kg + IVIg	half nBT062 2 mg/kg + IVIg	bs nBT062-natalizumab 2 mg/kg + IVIg
Vehicle control		0.4045 (ns)	0.0022 (**)	0.0018 (**)	0.0018 (**)	0.7891 (ns)	0.0018 (**)	0.0018 (**)	0.0018 (**)	0.5730 (ns)	0.0132 (*)	0.0018 (**)	0.0018 (**)	0.3726 (ns)	0.9787 (ns)	0.0277 (*)	0.2864 (ns)	0.4710 (ns)
IVIg			0.0025 (**)	0.0025 (**)	0.0025 (**)	0.3094 (ns)	0.0025 (**)	0.0025 (**)	0.0025 (**)	0.5918 (ns)	0.0079 (**)	0.0025 (**)	0.0025 (**)	0.9528 (ns)	0.1396 (ns)	0.0271 (*)	0.4479 (ns)	0.9400 (ns)
WT nBT062 4 mg/kg				0.3173 (ns)	0.3173 (ns)	0.0064 (**)	>0.9999 (ns)	0.3173 (ns)	0.9372 (ns)	0.0018 (**)	0.9372 (ns)	0.3173 (ns)	0.6071 (ns)	0.0026 (**)	0.0082 (**)	0.5203 (ns)	0.0127 (*)	0.0044 (**)
stable nBT062 4mg/kg					>0.9999 (ns)	0.0018 (**)	>0.9999 (ns)	>0.9999 (ns)	0.3173 (ns)	0.0018 (**)	0.3173 (ns)	>0.9999 (ns)	0.1343 (ns)	0.0026 (**)	0.0027 (**)	0.1343 (ns)	0.0018 (**)	0.0018 (**)
half nBT062 4 mg/kg						0.0018 (**)	>0.9999 (ns)	>0.9999 (ns)	0.3173 (ns)	0.0018 (**)	0.3173 (ns)	>0.9999 (ns)	0.1343 (ns)	0.0026 (**)	0.0027 (**)	0.1343 (ns)	0.0018 (**)	0.0018 (**)
bs nBT062-natalizumab 4mg/kg							0.0018 (**)	0.0018 (**)	0.0018 (**)	0.5670 (ns)	0.0143 (*)	0.0018 (**)	0.0018 (**)	0.2916 (ns)	0.8596 (ns)	0.0607 (ns)	0.5852 (ns)	0.4412 (ns)
WT nBT062 4 mg/kg + IVIg								>0.9999 (ns)	0.3173 (ns)	0.0018 (**)	0.3173 (ns)	>0.9999 (ns)	0.1343 (ns)	0.0026 (**)	0.0027 (**)	0.1343 (ns)	0.0018 (**)	0.0018 (**)
stable nBT062 4mg/kg + IVIg									0.3173 (ns)	0.0018 (**)	0.3173 (ns)	>0.9999 (ns)	0.1343 (ns)	0.0026 (**)	0.0027 (**)	0.1343 (ns)	0.0018 (**)	0.0018 (**)
half nBT062 4 mg/kg + IVIg										0.0018 (**)	0.9372 (ns)	0.3173 (ns)	0.6071 (ns)	0.0026 (**)	0.0027 (**)	0.4261 (ns)	0.004 (**)	0.0018 (**)
bs nBT062-natalizumab 4mg/kg + IVIg											0.0088 (**)	0.0018 (**)	0.0018 (**)	0.5952 (ns)	0.2077 (ns)	0.0273 (*)	0.2799 (ns)	0.9307 (ns)
WT nBT062 2 mg/kg												0.3173 (ns)	0.6071 (ns)	0.0084 (**)	0.0251 (*)	0.5203 (ns)	0.0143 (*)	0.0064 (**)
stable nBT062 2 mg/kg													0.1343 (ns)	0.0026 (**)	0.0027 (**)	0.1343 (ns)	0.0018 (**)	0.0018 (**)
half nBT062 2 mg/kg														0.0026 (**)	0.0027 (**)	0.7987 (ns)	0.0018 (**)	0.0018 (**)
bs nBT062-natalizumab 2 mg/kg															0.1368 (ns)	0.0348 (*)	0.2151 (ns)	0.9371 (ns)
WT nBT062 2 mg/kg + IVIg																0.0346 (*)	0.4644 (ns)	0.3702 (ns)
stable nBT062 2 mg/kg + IVIg																	0.0768 (ns)	0.0206 (*)
half nBT062 2 mg/kg + IVIg																		0.1642 (ns)
bs nBT062-natalizumab 2 mg/kg + IVIg																		

ns= not significant
important testing
