# Supplementary material for: Functional relevance of in vivo half antibody exchange of an IgG4 therapeutic antibody-drug conjugate
Source: PLoS One. 2018 Apr 19;13(4):e0195823. doi: 10.1371/journal.pone.0195823 (PMC5908158; doi:10.1371/journal.pone.0195823)
Supplement: S2 Table — (PDF) [file pone.0195823.s005.pdf]

S2 Table. Numbers of animals used, euthanized, and the cause of death for all animals.

Group ID	Therapy	Dose Level [mg/kg/day]	Last Day of Group	Max. Median BWL [%] (Day) [1]	Overall Survival Rate [2]	Euthanasia for Tumor-Related Reasons (Day)	Adjusted Survival Rate [3]	Other Deaths / Euthanasia (Day)
1	Vehicle Control	10 ml/kg	51	1.0 (2)	0 / 5 (0%)	5 x ATV > 2000 mm³ (23,27,27,44,51)	100%	-
2	Intratect	10 ml/kg	37	0.3 (2)	0 / 5 (0%)	4 x ATV > 2000 mm³ (23,30,37,37)<br>1 x accessory tumor (23)	100%	-
3	WT BT062-DM4	4	86	1.9 (2)	4 / 5 (80%)	-	80%	1 x labored breathing (48); Necropsy
4	Stable BT062-DM4	4	86	0.3 (2)	5 / 5 (100%)	-	100%	-
5	Half BT062-DM4	4	86	n.r.	5 / 5 (100%)	-	100%	-
6	Bispecific BT062-natalizumab-DM4	4	71	n.r.	0 / 5 (0%)	4 x ATV > 2000 mm³ (27,37,41,71)	80%	1 x ongoing BWL >2 days (8)
7	Intratect //<br>WT BT062-DM4	10 ml/kg //<br>4	86	0.6 (27)	5 / 5 (100%)	-	100%	-
8	Intratect //<br>Stable BT062-DM4	10 ml/kg //<br>4	86	1.5 (2)	5 / 5 (100%)	-	100%	-
9	Intratect //<br>Half BT062-DM4	10 ml/kg //<br>4	86	n.r.	4 / 5 (80%)	1 x ATV > 2000 mm³ (76)	100%	-
10	Intratect //<br>Bispecific BT062-natalizumab-DM4	10 ml/kg //<br>4	44	1.3 (2)	0 / 5 (0%)	5 x ATV > 2000 mm³ (23,27,34,37,44)	100%	-
11	WT BT062-DM4	2	86	n.r.	4 / 5 (80%)	1 x ATV > 2000 mm³ (37)	100%	-
12	Stable BT062-DM4	2	86	n.r.	5 / 5 (100%)	-	100%	-
13	Half BT062-DM4	2	86	n.r.	3 / 5 (60%)	2 x ATV > 2000 mm³ (79,83)	100%	-
14	Bispecific BT062-natalizumab-DM4	2	37	n.r.	0 / 5 (0%)	4 x ATV > 2000 mm³ (27,27,37,37)<br>1 x ulcerating tumor (20)	100%	-
15	Intratect //<br>WT BT062-DM4	10 ml/kg //<br>2	48	0.7 (2)	0 / 5 (0%)	5 x ATV > 2000 mm³ (23,27,44,48,48)	100%	-
16	Intratect //<br>Stable BT062-DM4	10 ml/kg //<br>2	86	1.9 (2)	3 / 5 (60%)	2 x ATV > 2000 mm³ (23,55)	100%	-
17	Intratect //<br>Half BT062-DM4	10 ml/kg //<br>2	76	9.4 (41)	0 / 5 (0%)	5 x ATV > 2000 mm³ (23,30,37,55,76)	100%	-
18	Intratect //<br>Bispecific BT062-natalizumab-DM4	10 ml/kg //<br>2	48	1.1 (30)	0 / 5 (0%)	5 x ATV > 2000 mm³ (16,23,27,34,48)	100%	-
