# Supplementary material for: Functional relevance of in vivo half antibody exchange of an IgG4 therapeutic antibody-drug conjugate
Source: PLoS One. 2018 Apr 19;13(4):e0195823. doi: 10.1371/journal.pone.0195823 (PMC5908158; doi:10.1371/journal.pone.0195823)
Supplement: S1 File — (PDF) [file pone.0195823.s006.pdf]

Fig 3A: Antibody binding to BaF3-hCD138 cells measured by flow cytometry [MFI values]

	WT nBT062		Stable nBT062		half nBT062		bs nBT062-natalizumab		natalizumab	
**Ab conc.**	*Mean*	*SD*	*Mean*	*SD*	*Mean*	*SD*	*Mean*	*SD*	*Mean*	*SD*
**0.0000005**	13606.5	31.5	13313.0	395.0	14424.7	491.6	12058.3	543.9	139.0	5.7
**1.67E-07**	13677.7	469.2	12798.0	213.9	13206.0	192.7	10339.0	639.1	63.3	1.6
**5.56E-08**	13061.0	438.4	12421.7	215.1	12703.7	370.7	8437.3	791.1	33.8	0.6
**1.85E-08**	13003.3	496.9	12234.0	270.4	12270.0	218.8	5822.7	616.4	23.5	0.6
**6.17E-09**	12973.0	479.8	11982.0	113.1	11900.7	167.5	2974.0	348.9	20.1	1.2
**2.06E-09**	11055.3	229.9	9653.0	262.0	8831.3	536.7	1211.7	115.4	18.9	0.6
**6.86E-10**	6358.3	53.2	5139.0	167.8	4237.3	429.5	489.7	47.8	18.0	0.0
**2.29E-10**	2798.0	78.7	2229.7	94.2	1729.3	185.1	190.7	20.7	18.4	0.6
**7.62E-11**	1206.7	24.5	950.3	52.9	692.7	94.6	80.1	6.2	18.0	1.1
**2.54E-11**	577.7	16.9	475.0	25.0	302.0	30.8	41.5	2.4	18.4	0.6
**8.47E-12**	305.3	14.3	256.7	5.8	152.3	16.2	28.6	0.6	18.4	0.6
**2.82E-12**	200.7	6.1	160.0	7.3	93.2	9.3	23.5	0.6	18.0	0.0

Fig 3B: Antibody binding to Jurkat cells measured by flow cytometry [MFI values]

	WT nBT062		Stable nBT062		half nBT062		bs nBT062-natalizumab		natalizumab	
**Ab conc.**	*Mean*	*SD*	*Mean*	*SD*	*Mean*	*SD*	*Mean*	*SD*	*Mean*	*SD*
**0.0000005**	30.8	0.0	44.1	1.6	56.1	1.6	6521.7	69.7	5033.7	112.7
**1.67E-07**	29.1	0.6	35.9	1.0	42.4	1.0	5787.7	103.3	4640.0	68.7
**5.56E-08**	28.2	0.0	31.7	0.6	36.0	2.8	5436.0	100.5	4413.7	42.7
**1.85E-08**	28.2	0.0	29.1	1.2	30.4	0.6	5035.7	42.4	4328.0	11.2
**6.17E-09**	28.2	1.0	28.6	0.6	29.1	0.6	4604.7	62.8	4269.0	11.2
**2.06E-09**	28.2	0.0	27.4	0.6	27.4	0.6	3206.3	62.4	4152.0	109.1
**6.86E-10**	27.4	0.6	27.0	1.0	26.1	0.6	1498.3	40.6	2837.7	434.6
**2.29E-10**	25.7	0.0	26.6	0.6	25.3	1.2	633.7	7.4	1265.7	257.9
**7.62E-11**	25.3	0.6	25.3	0.6	24.4	1.1	273.7	6.5	494.0	93.5
**2.54E-11**	27.4	2.4	24.8	0.6	24.4	0.0	129.0	1.6	221.0	37.0
**8.47E-12**	25.3	0.6	24.8	0.6	24.4	0.0	74.9	1.6	113.6	15.2
**2.82E-12**	24.8	0.6	24.8	0.6	24.8	0.6	56.1	0.6	77.5	9.1

Fig 4B: Flow cytometric internalization analysis [MFI values]

	WT nBT062		Stable nBT062		Half nBT062		bs nBT062-natalizumab + 50x natalizumab		natalizumab-Dy488 + 50x natalizumab	
**Conditions**	**Mean**	**SD**	**Mean**	**SD**	**Mean**	**SD**	**Mean**	**SD**	**Mean**	**SD**
**1. 4°C; 0.5 h; - Trypsin**	7037.7	330.4	7326.1	308.1	7751.1	382.2	3865.9	122.5	61.7	6.4
**2. 4°C; 0.5 h; + Trypsin**	75.4	7.5	77.8	12.0	74.9	11.2	353.6	44.8	75.3	4.1
**3. 37°C; 24 h; - Trypsin**	10476.1	454.9	11022.9	589.5	11198.4	507.6	10236.0	1193.1	140.6	35.9
**4. 37°C; 24 h; + Trypsin**	3975.9	235.3	4278.4	26.2	4220.9	220.4	5889.7	458.3	141.6	35.4

T-Test Comparison	**P-value**
WT nBT062 3. vs 4.	<0.0001
WT nBT062 1. vs 4.	<0.0001
WT nBT062 1. vs 3.	<0.0001
Stable nBT062 1. vs 3.	<0.0001
Half nBT062 1. vs 3.	<0.0001
bs nBT062-natalizumab 1. vs 3.	<0.0001
bs nBT062-natalizumab 1. vs 4.	<0.0001
Half nBT062 1. vs bs nBT062-natalizumab 1.	<0.0001
Half nBT062 4. vs bs nBT062-natalizumab 4.	0.0007
Half nBT062 3. vs bs nBT062-natalizumab 3.	0.0231

Fig 5: Cytotoxicity

	relative cytotoxicity	
	**Mean [%]**	**SD [%]**
**WT nBT062-DM4**	100.0	0.00
**Stable nBT062-DM4**	126.6	13.36
**Half nBT062-DM4**	84.6	15.39
**bs nBT062-natalizumab-DM4**	52.2	12.03
**bs nBT062-natalizumab-DM4 + 50x natalizumab**	31.9	3.92
**50x natalizumab**	0.0	0.00
**WT nBT062-DM4 + 50x natalizumab**	145.0	45.85

**T-Test**	**P-value**
WT nBT062-DM4 vs bs nBT062-natalizumab-DM4	0.0302
WT nBT062-DM4 vs bs nBT062-natalizumab-DM4 + 50x	0.0017
Half nBT062-DM4 vs bs nBT062-natalizumab-DM4 + 50x natalizumab	0.0285

Fig 6: Relative median tumor growth [in %]

**Days after randomization**	**Control**	**IVIg 10ml/kg/week**	**WT nBT062-DM4 4mg/kg/week**	**Stable nBT062-DM4 4mg/kg/week**	**Half nBT062-DM4 4mg/kg/week**	**bispecific nBT062-natalizumab-DM4 4mg/kg/week**	**WT nBT062-DM4 4mg/kg/week + IVIg**	**Stable nBT062-DM4 4mg/kg/week + IVIg**	**Half nBT062-DM4 4mg/kg/week + IVIg**	**bispecific nBT062-natalizumab-DM4 4mg/kg/week + IVIg**
0	100.0	100.0	100.0	100.0	100.0	100.0	100.0	100.0	100.0	100.0
2	167.3	195.1	191.3	153.2	182.3	193.6	212.0	161.3	184.0	203.9
6	301.6	342.4	109.1	77.6	89.3	253.4	152.0	81.7	119.0	372.3
9	408.3	469.9	42.2	33.4	39.9	409.4	35.3	44.1	64.0	481.5
13	482.0	575.5	0.2	0.2	15.9	506.8	16.9	0.2	18.1	625.1
16	725.6	956.0	0.1	0.1	0.1	629.0	0.2	0.2	14.5	949.6
20	759.0	1160.0	0.1	0.1	0.1	712.2	0.1	0.1	0.1	1080.1
23	1027.4	1279.9	0.1	0.1	0.1	676.7	0.1	0.1	0.1	1246.7
27	1826.1	2244.5	0.1	0.1	0.1	1051.1	0.1	0.1	0.1	1733.9
30	2197.8	2421.6	0.1	0.1	0.1	1172.4	0.1	0.1	0.1	1879.7
34	2303.7	2421.6	0.1	0.1	0.1	1304.3	0.1	0.1	0.1	2058.8
37	2303.7	2719.2	0.1	0.1	0.1	1517.9	0.1	0.1	0.1	2621.1
41	2388.1		0.1	0.1	0.1	1488.7	0.1	0.1	0.1	2833.7
44	2775.0		0.1	0.1	0.1	1488.7	0.1	0.1	0.1	3411.7
48	3050.9		0.1	0.1	0.1	1488.7	0.1	0.1	0.1	
51	3172.5		0.1	0.1	0.1	1488.7	0.1	0.1	0.1	
55			0.1	0.1	0.1	1488.7	0.1	0.1	0.1	
58			0.1	0.1	0.1	1488.7	0.1	0.1	0.1	
64			0.1	0.1	0.1	1853.3	0.1	0.1	0.1	
71			0.1	0.1	0.1	3592.2	0.1	0.1	0.1	
76			0.1	0.1	0.1		0.1	0.1	0.1	
79			0.1	0.1	0.1		0.1	0.1	0.1	
83			0.1	0.1	0.1		0.1	0.1	0.1	
86			0.1	0.1	0.1		0.1	0.1	0.1	

**Days after randomization**	**WT nBT062-DM4 2mg/kg/week**	**Stable nBT062-DM4 2mg/kg/week**	**Half nBT062-DM4 2mg/kg/week**	**bispecific nBT062-natalizumab-DM4 2mg/kg/week**	**WT nBT062-DM4 2mg/kg/week + IVIg**	**Stable nBT062-DM4 2mg/kg/week + IVIg**	**Half nBT062-DM4 2mg/kg/week + IVIg**	**bispecific nBT062-natalizumab-DM4 2mg/kg/week + IVIg**
0	100.0	100.0	100.0	100.0	100.0	100.0	100.0	100.0
2	158.4	202.5	189.9	177.1	219.9	237.2	219.1	215.7
6	154.4	125.4	94.8	294.2	261.4	230.2	233.5	322.3
9	96.2	38.8	59.4	439.3	489.5	217.4	324.6	402.4
13	42.2	0.1	30.9	709.8	511.6	76.4	403.2	497.2
16	33.1	0.1	0.2	843.7	752.6	44.8	565.3	805.6
20	0.2	0.1	0.2	1096.6	655.5	0.1	595.8	1128.4
23	0.2	0.1	0.1	1203.5	607.7	0.1	758.3	1249.2
27	18.4	0.1	0.1	2028.2	996.7	26.7	1293.7	1913.1
30	0.2	0.1	0.1	2062.8	1040.4	0.1	1293.7	2041.1
34	0.2	0.1	0.1	2185.0	1412.9	0.1	1293.7	2445.7
37	0.2	0.1	0.1	2610.8	1846.2	0.1	1338.2	2498.8
41	0.1	0.1	0.1		2124.8	0.1	1494.5	2735.4
44	0.2	0.1	0.1		2496.6	0.1	1574.0	2747.2
48	0.2	0.1	0.2		2673.3	0.1	1785.9	2860.9
51	0.2	0.1	0.2			0.1	1840.0	
55	0.2	0.1	0.2			0.1	1965.0	
58	0.2	0.1	0.2			0.1	1965.0	
64	0.2	0.1	0.2			0.1	1965.0	
71	0.2	0.1	0.2			0.1	2062.6	
76	0.2	0.1	0.2			0.1	2966.4	
79	0.2	0.1	0.2			0.1		
83	0.2	0.1	0.2			0.1		
86	0.2	0.1	0.1			0.1		

Fig 7:
see S1 Table + S2 Table

S3 Fig A: Cytotoxicity of nBT062-DM4 variants to NCI-H929 cells [fractions of viable cells]

**concentration**	**WT nBT062-DM4**		**Stable nBT062-DM4**		**Half nBT062-DM4**		**bs nBT062-natalizumab-DM4+50x natalizumab**	
	Mean	SD	Mean	SD	Mean	SD	Mean	SD
4.00E-09	0.057	0.027	0.041	0.016	0.028	0.022	-0.010	0.038
1.00E-09	0.021	0.020	0.029	0.012	0.000	0.017	0.191	0.116
4.00E-10	-0.007	0.010	-0.006	0.005	0.000	0.015	0.899	0.065
2.00E-10	0.002	0.007	-0.002	0.004	0.066	0.030	1.022	0.032
1.00E-10	0.438	0.280	0.092	0.049	0.870	0.097	1.081	0.032
8.00E-11	0.697	0.011	0.659	0.122	0.827	0.121	0.995	0.083
6.40E-11	0.909	0.184	0.809	0.054	0.844	0.087	0.907	0.115
3.20E-11	0.894	0.109	0.826	0.123	0.917	0.050	0.908	0.238
1.60E-11	0.932	0.225	0.988	0.033	0.948	0.173	1.003	0.192
0	1.000	0.000	1.000	0.000	1.000	0.000	1.000	0.000

S3 Fig B: Cytotoxicity of nBT062-DM4 variants to Jurkat cells [fractions of viable cells]

**concentration**	**WT nBT062-DM4**		**Stable nBT062-DM4**		**Half nBT062-DM4**		**bs nBT062-natalizumab-DM4+50x natalizumab**	
	Mean	SD	Mean	SD	Mean	SD	Mean	SD
4.00E-09	0.772	0.026	0.991	0.130	0.715	0.173	0.967	0.064
1.00E-09	0.835	0.051	0.942	0.095	0.867	0.057	0.986	0.115
4.00E-10	0.926	0.019	0.977	0.103	0.946	0.027	1.077	0.088
2.00E-10	0.920	0.039	1.077	0.087	0.916	0.083	1.065	0.069
1.00E-10	0.879	0.047	0.925	0.131	0.922	0.057	1.019	0.072
8.00E-11	0.824	0.008	1.022	0.115	0.899	0.097	1.078	0.084
6.40E-11	0.907	0.018	0.991	0.085	0.888	0.151	1.062	0.095
3.20E-11	0.817	0.018	0.917	0.120	0.812	0.063	1.032	0.096
1.60E-11	0.894	0.041	1.034	0.138	0.937	0.138	0.944	0.138
0	1.000	0.000	1.000	0.000	1.000	0.000	1.000	0.000
